# BICD1 mediates HIF1α nuclear translocation in mesenchymal stem cells during hypoxia adaptation

**DOI:** 10.1038/s41418-018-0241-1

**Published:** 2018-11-21

**Authors:** Hyun Jik Lee, Young Hyun Jung, Ji Young Oh, Gee Euhn Choi, Chang Woo Chae, Jun Sung Kim, Jae Ryong Lim, Seo Yihl Kim, Sei-Jung Lee, Je Kyung Seong, Ho Jae Han

**Affiliations:** 1 Department of Veterinary Physiology, College of Veterinary Medicine, Research Institute for Veterinary Science, and BK21 PLUS Program for Creative Veterinary Science Research, Seoul National University, Seoul, 08826 Republic of Korea; 2 Department of Agricultural Biotechnology, Animal Biotechnology Major, and Research Institute of Agriculture and Life Science, Seoul National University, Seoul, 08826 Republic of Korea; 3 Department of Pharmaceutical Engineering, Daegu Haany University, Gyeongsan, 38610 Republic of Korea; 4 Laboratory of Developmental Biology and Genomics, Research Institute for Veterinary Science, BK21 PLUS Program for Creative Veterinary Science Research, College of Veterinary Medicine, and Korea Mouse Phetnotyping Center (KMPC), Seoul National University, Seoul, 08826 Republic of Korea

**Keywords:** Cell biology, Molecular biology

## Abstract

Hypoxia inducible factor 1α (HIF1α) is a master regulator leading to metabolic adaptation, an essential physiological process to maintain the survival of stem cells under hypoxia. However, it is poorly understood how HIF1α translocates into the nucleus in stem cells under hypoxia. Here, we investigated the role of a motor adaptor protein Bicaudal D homolog 1 (BICD1) in dynein-mediated HIF1α nuclear translocation and the effect of BICD1 regulation on hypoxia adaptation and its therapeutic potential on human umbilical cord blood-derived mesenchymal stem cells (UCB-MSCs). In our results, silencing of *BICD1* but not *BICD2* abolished HIF1α nuclear translocation and its activity. BICD1 overexpression further enhanced hypoxia-induced HIF1α nuclear translocation. Hypoxia stimulated direct bindings of HIF1α to BICD1 and the intermediate chain of dynein (Dynein IC), which was abolished by *BICD1* silencing. Akt inhibition reduced the binding of BICD1 to HIF1α and nuclear translocation of HIF1α. Conversely, Akt activation or *GSK3β* silencing further enhanced the hypoxia-induced HIF1α nuclear translocation. Furthermore, *BICD1* silencing abolished hypoxia-induced glycolytic reprogramming and increased mitochondrial ROS accumulation and apoptosis in UCB-MSCs under hypoxia. In the mouse skin wound healing model, the transplanted cell survival and skin wound healing capacities of hypoxia-pretreated UCB-MSCs were reduced by *BICD1* silencing and further increased by *GSK3β* silencing. In conclusion, we demonstrated that BICD1-induced HIF1α nuclear translocation is critical for hypoxia adaptation, which determines the regenerative potential of UCB-MSCs.

## Introduction

Hypoxia inducible factor 1 (HIF1) is a master regulator for hypoxia-stimulated metabolic adaptation through the induction of anaerobic glycolytic enzymes [1]. The HIF1-induced metabolic switch during hypoxia adaptation is a key physiological process to reduce the oxidative metabolism-dependent toxic ROS production and ATP demands [2–4]. This HIF1-induced metabolic reprogramming is required for maintaining the survival of stem cells under hypoxia [5, 6]. Under hypoxia, the alpha subunit of HIF1 (HIF1α) accumulates by the inactivation of von Hippel-Lindau tumor suppressor protein (pVHL)-mediated proteosomal degradation and dimerizes with HIF1β in the nucleus [7, 8]. Previous studies investigating the mechanism of HIF1α nuclear transport demonstrated that microtubule stabilization is an important factor for HIF1α nuclear translocation under hypoxia [9, 10]. In addition, it has been suggested that a microtubule motor protein, cytoplasmic dynein, mediates the microtubule association of HIF1α involved in its nuclear translocation [11]. These reports provide proof that dynein-mediated HIF1α nuclear transport is a key factor regulating the hypoxia adaptation under hypoxia with the low activity of pVHL [9, 11]. However, the mechanism for how hypoxia regulates dynein-mediated HIF1α nuclear translocation is poorly understood. Moreover, microtubule stabilizers and dynein inhibitors have a limitation in that they affect the general cell physiology as well as HIF1α. Therefore, an investigation into the control of the interaction between dynein and HIF1α will provide a new strategy for enhancing the hypoxia adaptation capacity of stem cells.

Bicaudal D (BICD) is an evolutionarily conserved motor adaptor protein between microtubule-associated motor proteins including dynein and their cargo for minus end-directed transport [12]. The N-terminal region of BICD is recruited to microtubule motor proteins and organelles [12, 13]. Meanwhile, the C-terminal region of BICD has a cargo binding domain involved in the transport of Rab6-dependent vesicles, viral genome and several kinds of proteins [14–16]. It has been reported that BICDs have physiological roles in various kinds of intracellular cargo transport and in the central positioning of the centrosome and nucleus in mammalian neuron and mitotic cells [17]. Splinter et al. [18] reported that BICD2 binds to nuclear pore complex (NPC) on the nuclear envelope in the G2 phase prior to mitosis, but it does not interact with Rab6. This indicates that BICD has mutually exclusive roles depending on the cellular physiological status. Noticeably, BICD2 directly binds to NPC, and a SUMO E3 ligase RanBP2, also known as a Nup358, is presented as a major interacting partner of BICD [18, 19]. RanBP2 is a major component of the cytoplasmic filament of NPC and closely associated with the nuclear translocation of transcription factors [20]. Although the role of BICD in HIF1α nuclear transport has not been reported yet, previous findings suggest a possibility that BICD could be a factor regulating dynein-mediated HIF1α nuclear translocation.

Human umbilical cord blood-derived mesenchymal stem cells (UCB-MSCs) are abundant non-embryonic multipotent stem cell sources that have multiple lineage differentiation potential and immune modulation capacity [21, 22]. Many stem cell studies have shown that mesenchymal stem cell (MSC)-based therapy is a promising therapeutic strategy for the treatment of inflammatory, ischemic and neurodegenerative diseases [23, 24]. However, the low survival rate of transplanted MSCs induced by oxidative stress is the greatest obstacle to MSC transplantation into the patients. Importantly, *HIF1A* mRNA expression is 32-fold higher in UCB-MSCs than in HEK cells under normoxia suggesting that UCB-MSCs have a great capacity for HIF1α-induced metabolic adaptation under hypoxia [25]. Indeed, hypoxia-preconditioned MSCs exhibit a high survival rate and therapeutic potential compared with normoxia-preconditioned MSCs [26–28]. Concerning that HIF1α can be stimulated by serum-activated Akt pathway, culture condition of the UCB-MSC can lead higher HIF1α expression level than other cells [29–32]. Therefore, an investigation into the regulation of HIF1α nuclear translocation for hypoxia adaptation is necessary to improve the therapeutic effect of MSC transplantation. To address this issue, we investigated the role of BICD in the nuclear translocation of HIF1α and determined the effects of BICD regulation on hypoxia adaptation and the regenerative potential of UCB-MSCs.

## Materials and methods

### Materials

The UCB-MSCs were acquired from Kang Stem Biotech (Seoul, Korea). Fetal bovine serum (FBS) and antibiotics were purchased from Hyclone (Logan, UT, USA) and Gibco (Grand Island, NY, USA), respectively. The reagents used in this study were purchased from Sigma-Aldrich (St. Louis, MO, USA) and are listed as follows: Ciliobrevin D (Sigma-Aldrich, #250401), nocodazole (Sigma-Aldrich, #M1404), wortmannin (Sigma-Aldrich, #W1628), MG-132 (Sigma-Aldrich, #M7449), and SC-79 (Sigma-Aldrich, #SML0749). The antibodies used in this study are listed as follows: anti-HIF1α (Abfrontier, Seoul, Korea, #YF-MA13455), anti-Lamin A/C (Santa Cruz Biotechnology, Dallas, TX, USA, #sc2068), anti-α-Tubulin (Abfrontier, #LF-PA0146), anti-intermediate chain of dynein (Dynein IC, Santa Cruz Biotechnology, #sc-66866), anti-BICD1 (Novus Biologicals, Littleton, CO, USA, #NBP1-78735), anti-BICD2 (Novus Biologicals, #NBP1-81488), anti-Importin α3 (Abfrontier, #YF-MA10506), anti-RanBP2 (Novus Biologicals, #NB120-2938), anti-β-Actin (Santa Cruz Biotechnology, #sc-47778), anti-p-GSK3β (Ser9, Santa Cruz Biotechnology, #sc-11757), anti-GSK3β (Santa Cruz Biotechnology, #sc-9166), anti-Cleaved caspase-3 (Cell Signaling Technology, Beverly, MA, USA, #9661) and anti-Caspase-9 (Santa Cruz Biotechnology, #sc-8355). The plasmids for pcDNA3.1/BICD1-c-eGFP and pcDNA3.1/c-eGFP were purchased from KomaBiotech, Seoul, Korea). mRNA primers for *HK1*, *LDHA*, *G6PD*, and *ACTB* were purchased from Bioneer (Daejeon, Korea). Small interfering RNAs (siRNAs) for *BICD1*, *BICD2*, *GSK3β*, and NT were purchased from Dharmacon (Lafayette, CO, USA).

### Cell cultivation and hypoxia treatment

The UCB-MSCs were cultured with α-minimum essential medium (α-MEM; Hyclone, #SH30265.01), 10% FBS and antibiotics at 37 ℃ with 5% CO_2_. Cells grown to 80% confluency were washed with phosphate-buffered solution (PBS; Hyclone, #SH30256). To reduce the effect of the serum, the UCB-MSCs were incubated with α-MEM with 5% Knockout™ serum replacement (SR; Gibco, #10828028) for 24 h. The SK-N-MC neuroblastoma cells were provided by the Korean Cell Line Bank (Seoul, Korea). The SK-N-MCs were cultured with high glucose Dulbecco’s essential medium (DMEM; Hyclone, #SH30243.01), 10% FBS and antibiotics at 37 ℃ with 5% CO_2_. For serum reduction, the SK-N-MCs were incubated with DMEM with 2% SR for 24 h prior to hypoxia treatment. BICD1 knock out (BICD1 KO) SK-N-MCs were established using CRISPR/Cas9 system. For the hypoxia treatment, a modular hypoxia incubation chamber (Billups-Rothenberg, Del Mar, CA, USA) was used. The hypoxia chamber was purged with hypoxic gas (0.5% O_2_, 5% CO_2_, and 94.5% N_2_) at a 5 L/min flow rate for 15 min and then incubated in a cell incubator at 37 ℃.

### Western blot analysis

The western blot analysis was performed essentially according to the protocol previously described using the indicated antibodies [33, 34]. Briefly, cells were washed in ice-cold PBS twice and harvested with a cell scraper. Pelleted samples were lysed with RIPA lysis buffer (Atto, Tokyo, Japan, #AE6500) with protease and phosphatase inhibitors. Cell debris was removed by centrifugation (13,000 × *g*, 4 ℃, 30 min). Protein determination was performed by a bichichoninic acid (BCA) quantification assay (Thermo Fisher, #23227). Laemelli sample buffer was added to the samples. Then, the protein samples were boiled at 100 ℃ for 5 min. Finally, 10 μg of protein samples were loaded into a 8–12% SDS-polyacrylamide gel and transferred to a polyvinylindene fluoride (PVDF) membrane. The membrane was washed with tris-buffered saline containing 0.2% Tween-20 {TBST; 150 mM NaCl, 10 mM Tris-HCl (pH 7.6), 0.1% Tween-20} three times. The membranes were blocked with 5% skim milk (Gibco, Grand Island, NY, USA, #232100) in TBST for 30 min. The membrane was incubated with a primary antibody solution (1:1,000 dilution) at 4 ℃ overnight. After washing with TBST three times, the membrane was incubated with anti-mouse or rabbit horseradish peroxidase (HRP)-conjugated secondary antibody solution (1:10,000 dilution) at room temperature for 2 h. Western blots were detected with a chemiluminescence detection kit (Advansta Inc., Menlo Park, CA, USA, #K-12045-D50). Protein bands were analyzed with the ImageJ software (developed by Wayne Rasband, National Institutes of Health, Bethesda, MD, USA; imagej.nih.gov./ij/). To blot the control proteins including β-Actin, Lamin A/C and α-Tubulin, membranes were incubated in stripping buffer (1.5% glycine, 0.2% SDS, 1% Tween-20, pH 2.2) for 30 min. For the preparation of the cytosolic and nuclear fractionized samples, the EzSubcell™ subcellular fractionation/extraction kit (Atto, Tokyo, Japan, #WSE-7421) was used. Cytosolic and nuclear samples for the western blot analysis were prepared according to the manufacturer’s instructions. Nuclear HIF1α expression levels in nuclear fractionized samples were normalized by Lamin A/C expression levels.

### Immunocytochemistry analysis

For the immunocytochemistry, UCB-MSCs were fixed with 4% paraformaldehyde (PFA; Lugen Sci, Seoul, Korea, #LGB-1175) for 10 min, and then incubated in 0.5% Tween-20 for 10 min. Cells were incubated with primary antibodies in PBS containing 0.1% Tween-20 (PBST; 1:100 dilution) for 2 h and washed with PBS three times. Cells were incubated with Alexa Fluor^™^ 488 or 555-conjugated secondary antibodies in PBST (1:100 dilution) for 1 h. Immunofluorescence stained samples were visualized by a super-resolution radial fluctuations (SRRF) imaging system (Andor Technology, Belfast, UK) [35]. Relative fluorescence intensities of HIF1α, HIF1α/BICD1 and HIF1α/Dynein IC were quantified with the ImageJ software. The co-localization rate of HIF1α with BICD1 was analyzed with the MetaMorph^™^ software (Universal Imaging, West Chester, PA, USA).

### Co-immunoprecipitation

Harvested UCB-MSCs were lysed with co-immunoprecipitation lysis buffer (20 mM Tris–HCl pH 8.0, 137 mM NaCl, 1% Nonidet P-40, and 2 mM EDTA) with a protease inhibitor and incubated for 30 min on ice. Protein concentrationf determination was performed by a BCA quantification assay (Thermo Fisher, #23225). Primary antibodies used in this study were immobilized with SureBeads™ Protein G magnetic beads (BioRad, Hercules, CA, USA, #161-4021). Immobilized magnetic beads were washed in PBST three times and then incubated with cell lysates for 6 h at 4 ℃. Beads were washed in PBST three times and incubated with elution buffer (20 mM glycine pH 2.0) for 5 min. 1 M phosphate buffer and Laemelli sample buffer were added to the samples. Then, the protein samples were boiled at 100 ℃ for 5 min. Protein analysis was performed by western blot analysis. Anti-mouse or rabbit IgG antibodies were used as a negative control.

### Transfection of siRNAs for gene silencing

Prior to normoxia or hypoxia treatment, UCB-MSCs were incubated with 25 nM of the indicated siRNAs and transfection reagent TurboFect™ (Thermo Fisher, Waltham, MA, USA, #R0531) for 24 h without antibiotics. The medium was changed to α-MEM with 5% SR and 1% antibiotics prior to the normoxia or hypoxia treatment. The sequences for the siRNAs indicated in this study are described in Table S1. We confirmed that the siRNA efficacies for BICD1, BICD2 and GSK3β were at least 70%. Non-targeting siRNA (NT) was used as a control siRNA.

### Transfection of plasmid DNA

Prior to the normoxia or hypoxia treatment, UCB-MSCs were incubated with a mixture of plasmid DNA (pcDNA3.1/BICD1-cEGFP or pcDNA3.1/cEGFP), Lipofectamine™ Stem transfection reagent (Thermo Fisher, #STEM0015) and α-MEM for 6 h. The medium was changed to α-MEM medium with 10% SR and 1% antibiotics and incubated for 18 h prior to the normoxia or hypoxia treatment.

### Polymerase chain reaction (PCR)

RNA samples were extracted using a commercial RNA extraction kit (TaKaRa, Otsu, Shiga, Japan, #9767). Then, 1 μg of RNA was reverse-transcribed with a reverse transcription-PCR premix (iNtRON Biotechnology, Seongnam, Korea, #25081). Reverse transcription was performed for 1 h at 45 ℃ followed by 5 min at 95 ℃. The cDNA samples were amplified with the mRNA primers indicated in this study and a TB™ Green Premix Ex Taq™ (TaKaRa, #RR420A). The relative mRNA expression levels of the target genes were quantified with double delta Ct analysis, and the data were normalized with the *ACTB* mRNA expression levels. Quantitative real-time PCR was performed as follows: 10 min at 95 ℃ for DNA polymerase activation and 50 cycles of 15 s at 94 ℃, 15 s at 55 ℃, and 30 s at 72 ℃. The identity and specificity of the amplified PCR product was validated by melting curve analysis. The sequences of the mRNA primers used in this study are described in Table S2.

### In situ proximity ligation assay (PLA)

HIF1α/BICD1 and HIF1α/Dynein IC interactions were detected in situ using Duolink™ II secondary antibodies and detection kits (Sigma–Aldrich, #DUO92001, #DUO92005, and #DUO92008) according to the manufacturer’s instructions. Briefly, PLA probes and primary antibodies against anti-HIF1α, anti-BICD1 and anti-Dynein IC were applied to fixed cells. Then, Duolink™ secondary antibodies were added. These secondary antibodies were ligated together to make a closed circle by the Duolink™ ligation solution if the antibodies were in close proximity ( < 40 nm). Polymerase and amplification buffer were added to amplify the positive signal (red dot) of exiting closed circle and detected by SRRF microscopy. DAPI was used for counterstaining of the nucleus.

### Measurement of HIF1 transcriptional activity

The transcriptional activity of HIF1 was assessed via a HIF1-responsive dual firefly/Renilla luciferase Cignal™ reporter assay system (Qiagen, Hilden, Germany, #CCS-007L). UCB-MSCs were seeded at a density of 4 × 10^5^ cells/well with a transfection mixture of 200 ng of Cignal™ reporter construct, 25 ng of siRNA and Lipofectamine™ Stem transfection reagent for 24 h according to the manufacturer’s instructions. Cells were incubated under normoxia or hypoxia condition for 24 h. The HIF1 reporter activity in UCB-MSCs was assessed using a dual luciferase reporter assay system (Promega, Madison, WI, USA, #E1910). The DLR assay was performed according to the manufacturer’s instructions. The luciferase activities of firefly and Renilla were measured using a luminometer (Victor3; Beckman Coulter, Fullerton, CA, USA)

### Measurements of the hexokinase activity and lactate production

The hexokinase colorimetric assay kit (Biovision, Mountain View, CA, USA, #K789) and lactate colorimetric assay kit (Biovision, #K607) were used to measure the hexokinase activity and lactate production in the UCB-MSCs. The assays were performed according to the manufacturer’s instructions. The activity of the hexokinase and cellular lactate levels in the UCB-MSCs were measured with a microplate reader at 450 nm for the hexokinase activity assay and at 570 nm for the lactate assay.

### Intracellular pH measurement

To measure the intracellular pH, we used the cell permeable pH-sensitive fluorescent probe BCECF-AM [2’,7’-Bis-(2-carboxyethyl)-5-(and-6)-carboxyfluorescein, acetoxymethyl ester; Thermo Fisher, #B1150]. After the hypoxia or normoxia treatment, cells were washed with PBS twice. Then, the cells were incubated in 2 μM of BCECF-AM in PBS and kept at 37 ℃ for 10 min. Cells were washed with PBS twice. The fluorescence intensity (excitation/emission = 485/535 nm) of the BCECF-AM-stained cells were measured with a luminometer (Victor3).

### Annexin V-FITC/ PI staining

Fluorescein isothiocyanate-conjugated annexin V (Annexin V-FITC) and propidium iodide (PI) staining analysis was performed with an Annexin V-FITC apoptosis detection kit (BD Bioscience, Franklin Lakes, NJ, USA, #556547). The analysis was performed according to the manufacturer’s instruction. After the normoxia or hypoxia treatment, UCB-MSCs (1 × 10^5^ cells) were suspended in binding buffer. Annexin V-FITC and PI were added to the samples and incubated for 15 min at room temperature. Apoptosis of the samples was measured with flow cytometry (Quanta SC; Beckman Coulter). The samples were analyzed by the flowing software 2 (developed by Perttu Terho, Turku, Finland). Annexin V-FITC-negative and PI-positive (Q1), Annexin V-FITC-positive and PI-positive (Q2), and Annexin V-FITC-positive and PI-negative (Q4) UCB-MSCs were considered as late apoptotic, apoptotic, early apoptotic, respectively. Annexin V-FITC-negative and PI-negative (Q3) UCB-MSCs were considered viable. The following formula was used to determine the percentage of apoptotic cells: Apoptotic cells (%) = Q1 + Q2 + Q4.

### Water soluble tetrazolium salt (WST-1) cell proliferation and viability assay

The proliferation and viability of the UCB-MSCs were determined with the WST-1 cell viability assay kit (EZ-Cytox™; Daeil Labservice, Seoul, Korea, #EZ-1000). The assay was performed according to the manufacturer’s instructions. Briefly, *BICD1*, *BICD2* or NT siRNA-transfected UCB-MSCs cultured in 96-well plates were treated with normoxia or hypoxia for 24 h. Cells were incubated in 10 μL of EZ-Cytox™ solution in 100 μL of medium for 30 min in a cell incubator at 37 ℃. Then, the absorbance was measured with a microplate spectrophotometer (Epoch 2™; BioTek, Winooski, VT, USA) at 450 nm.

### Trypan blue cell viability assay

The UCB-MSCs were washed twice with PBS, and then incubate with a 0.05% trypsin and 0.5 mM EDTA solution to detach the cells. Soybean trypsin inhibitor was added to cell suspension solution to quench trypsin. Cell suspension solution was centrifugated 1,500 × *g* for 5 min. Cell pellet was suspended with 0.4% trypan blue (Sigma–Aldrich, #T6146) in PBS to stain the dead cells. Typan blue-stained and –unstained cells were counted by using a Petroff-Hausser counting chamber (Hausser Scientific, Horsham, PA, USA). Cell viability = [{1-(Trypan blue-stained cell number/total cell number)} × 100].

### Measurements of intracellular ROS, mitochondrial ROS, and mitochondrial membrane potential

The CM-H_2_DCFDA (Thermo Fisher, #C6821), MitoSOX Red^™^ (Thermo Fisher, #M36008) and tetramethylrhodamine, ethyl ester (TMRE; Sigma-Aldrich, #87917) was used for measure the intracellular ROS, mitochondrial ROS and mitochondrial membrane potential, respectively. The detailed protocols were previously described [33, 34]. The fluorescence intensity of CM-H_2_DCFDA was measured with a luminometer at an excitation and emission wavelength of 485 and 535 nm and those of MitoSOX Red™ and TMRE were measured at an excitation and emission wavelength of 530 and 580 nm.

### Mitochondrial stress test and glycolysis stress test assays

The oxygen consumption rate (OCR) under mitochondrial stress test assay and the extracellular acidification rate (ECAR) under glycolysis stress test assay were performed using the Seahorse XF24 Extracellular Flux Analyzer (Agilent Technologies, Santa Clara, CA, USA). Mitochondrial stress and glycolysis stress test assays were performed using XF Cell Mito Stress Test kit (Agilent Technologies, #103015-100) and XF Glycolysis Stress Test Kit (Agilent Technologies, #103020-100), respectively. The assays were performed according to the manufacturer’s instructions. The UCB-MSCs (1 × 10^4^ cells/well) were cultured in XF24 cell culture microplate (Agilent Technologies, #100777-004). For mitochondrial stress test assay, oligomycin (1 μM), carbonyl cyanide-4-(trifluoromethoxy)phenylhydrazone (FCCP, 0.5 μM) and antimycin A and rotenone mixture (0.5 μM) were treated to cell culture plate to determine the mitochondrial respiration including basal respiration, maximal respiration and spare respiratory capacity. For glycolysis stress test assay, D-glucose (10 mM), oligomycin (1 μM) and 2-deoxy-D-glucose (50 mM) were treated to cell culture plate to determine the glycolytic flux including glycolysis, glycolytic capacity and glycolytic reserve.

### PI staining with live-cell imaging

Cells were transfected with NT or *BICD1* siRNA for 24 h and then incubated with 0.1 μg/mL of PI-supplied SR media in a live-cell imaging chamber (Tokai, Tokyo, Japan). Normoxic (5% CO_2_, 21% O_2_, and 74% N_2_) or hypoxic (5% CO_2_, 0.5% O_2_, and 94.5% N_2_) gas was supplied to chamber for 48 h. Differential interference contrast images and red fluorescence protein images were acquired over 72 h at 6 h intervals with an Olympus IX81-ZDC zero drift microscope and a Cascade 512 B camera (Roper Scientific, Tucson, AZ, USA). The number of PI-positive cells was determined with the ImageJ software.

### Mouse skin wound healing model

All procedures for the mouse skin wound healing model were performed following the National Institutes of Health Guidelines for Humane Treatment of Animals and approved by the Institutional Animal Care and Use Committee of Seoul National University (SNU-161128-6). Eight-week-old male Institute of Cancer Research (ICR) mice were used. All mice were anesthetized with a mixture of Alfaxan™ (80 mg/kg, Jurox Pty Ltd, Rutheford, Australia) and xylazine HCl (10 mg/kg, Rompun™, Bayer, Leverkusen, Germany). The authors who have a doctor of veterinary medicine license granted by the Ministry of Agriculture and Forestry of Republic of Korea performed wound surgery. The procedure for the mouse skin wound healing surgery was performed as previously described [33]. Briefly, the back of an anesthetized mouse was shaved and scrubbed with an organic iodine solution and 70% ethanol solution for disinfection during the surgery. A wound in the back skin was made by using a 6 mm diameter circular biopsy punch. *BICD1*, *GSK3β* or NT siRNA-transfected UCB-MSCs were pretreated with normoxia or hypoxia for 24 h. Then, 1 × 10^6^ UCB-MSCs were injected into the dermis intradermally at three sites around each wound. Experimental mice groups were divided into the seven groups: mice given vehicle (group 1, *n* = 7); mice given NT siRNA-transfected UCB-MSCs (group 2, *n* = 7); mice given NT siRNA-transfected UCB-MSCs with hypoxia pretreatment (group 3, *n* = 7); mice given *BICD1* siRNA-transfected UCB-MSCs with hypoxia pretreatment (group 4, *n* = 7); mice given *BICD1* siRNA-transfected UCB-MSCs (group 5, *n* = 7); mice given *GSK3β* siRNA-transfected UCB-MSCs with hypoxia pretreatment (group 6, *n* = 7); and mice given *GSK3β* siRNA-transfected UCB-MSCs. All gross images were acquired at post-injection days 0, 4, 7 and 10. After the surgery, wounds were covered with Tegaderm™ (3M, London, Canada). The wound closure rate was analyzed by the ImageJ software. All mice were euthanized at post-injection day 10. Acquired skin samples were fixed with 4% PFA and then dehydrated with 20% and 30% sucrose solution. Dehydrated skin samples were embedded in optimum cutting temperature (O.C.T.) compound (Sakura Finetek, CA, USA, #4583) and stored in a deep freezer kept at –80 ℃. Frozen skin samples were sectioned to a 10 μm thickness using a cryostat (Leica CM 1520, Leica, Wetzlar, Germany) and mounted on silane-coated slides (Muto Pure Chemicals, Tokyo, Japan, #5116-20F). The vessel intensities in the skin wounds were analyzed with the ImageJ software.

### Hematoxylin and eosin (H & E) staining

Skin-mounted slides were fixed with 4% PFA for 5 min, and then stained with H & E for 5 min. Samples were washed with 70%, 95%, and 100% ethanol three times and then incubated in xylene for 5 min. All images were acquired by light microscopy. Histological evaluation and reepithelization scoring were performed in a blind fashion. The reepithelization of the wound site was evaluated according to a criteria described in Table S3.

### Immunohistochemistry

Skin samples on slides were fixed in 80% acetone solution for 20 min. Slides were washed in PBS and incubated in 5% normal goat serum (Sigma-Aldrich, #566380) for 30 min. Samples were incubated with primary antibody in PBS containing 0.2% Tween-20 (PBST) for 2 h. After washing with PBST, samples were incubated with Alexa Fluor™ 488 or 555-conjugated secondary antibodies in PBST (1:100 dilution) for 1 h. Immunohistochemistry images were acquired by Eclipse Ts2™ fluorescence microscopy (Nikon, Tokyo, Japan). All images were analyzed with the MetaMorph™ software.

### Statistical analysis

Quantitative data are shown as the mean ± standard error of the mean (S.E.M). Differences among experimental groups were analyzed with the analysis of variance. Comparing the means of treatment groups with that of the control was conducted with the Student’s *t*-test. *p* < 0.05 was considered statistically significant.

## Results

### Hypoxia-stimulated HIF1α nuclear translocation is dependent on BICD1

**Fig. 1:**
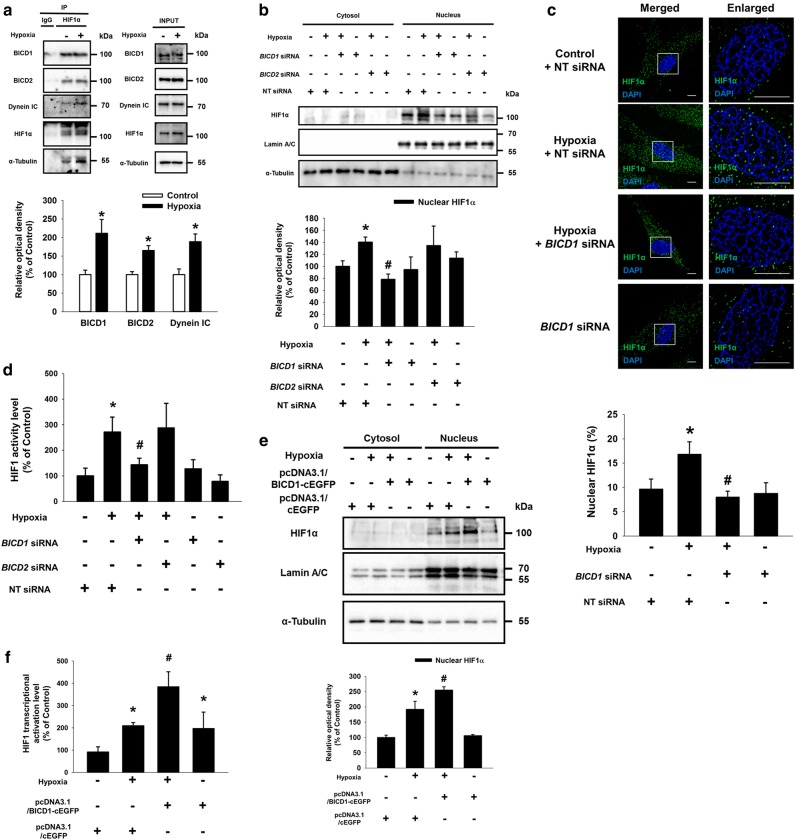
Role of BICD1 in dynein-mediated HIF1α nuclear translocation in UCB-MSCs. **a** Co-immunoprecipitation of BICD1, BICD2, Dynein IC, and α-Tubulin with IgG and HIF1α antibodies were shown in left panel. Total protein expressions in lysate were shown in right panel. *n* = 3. *p < 0.05 vs. normoxia control. **b–d** Cells were transfected with *BICD1*, *BICD2* or NT siRNA prior to hypoxia incubation for 24 h. **b** HIF1α, Lamin A/C, and α-Tubulin in cytosolic and nuclear fractionized samples were detected by western blot. *n* = 3. **c** Cells were immunostained with HIF1α-specific antibody. Scale bars are 8 μm (Magnification, × 1,000). *n* = 5. **d** HIF1 activity was measured by dual luciferase reporter assay. *n* = 6. *p < 0.05 vs. normoxia control with NT siRNA transfection, ^#^*p* < 0.05 vs. hypoxia with NT siRNA transfection. **e**, **f** Cells were transfected with plasmid vector for 24 h prior to hypoxia treatment for 24 h. **e** HIF1α, Lamin A/C, and α-Tubulin in cytosolic and nuclear fractionized samples were detected by western blot. *n* = 3. **f** HIF1 activity was measured by dual luciferase reporter assay. *n* = 7. HIF1α expression in nuclear fractionized samples was normalized by Lamin A/C. Quantitative data are presented as a mean ± S.E.M. All blot and immunofluorescence images are representative. *p < 0.05 vs. normoxia control with pcDNA3.1/cEGFP vector transfection, ^#^*p* < 0.05 vs. hypoxia with pcDNA3.1/cEGFP vector transfection

We investigated the role of microtubules and cytosolic dynein in the nuclear translocation of HIF1α in UCB-MSCs under hypoxia. Our data revealed that hypoxia-induced HIF1α nuclear translocation was suppressed by the nocodazole and ciliobrevin D pretreatment (Supplementary Fig. S1A–C). And, the ciliobrevin D pretreatment with hypoxia increased the cleaved caspase-9 protein expression and the percentage of Annexin V-positive cells compared to the hypoxia treatment alone (Supplementary Fig. S2A, B). These results indicate that hypoxia-induced HIF1α nuclear translocation is dependent on microtubule stability and cytosolic dynein activity, critical for the survival of UCB-MSCs under hypoxia. Next, we investigated whether BICD1 and BICD2 interact with dynein in UCB-MSCs with or without hypoxia. We found that hypoxia stimulated the binding of HIF1α to BICD1, BICD2, Dynein IC and α-Tubulin (Fig. 1a). Meanwhile, ciliobrevin D pretreatment did not affect the binding of α-Tubulin to BICD1 and BICD2 (Supplementary Fig. S3). siRNA transfection of *BICD1* but not *BICD2* inhibited the HIF1α nuclear translocation and activity (Fig. 1b–d). And, the hypoxia-induced HIF1α nuclear translocation and activity were further increased by pcDNA3.1/BICD1-cEGFP plasmid transfection (Fig. 1e, f and Supplementary Fig. S4). To detect the prolyl hydroxylated form (Hyp402 and Hyp564) of HIF1α, we treated MG132 to UCB-MSCs under normoxia or hypoxia [36]. Our results showed that *BICD1* silencing or overexpression did not affect the hypoxia-reduced Hyp402 and Hyp564 of HIF1α levels (Supplementary Fig. S5A, B).

We investigated the effect *BICD1* or *BICD2* silencing on HIF1α nuclear translocation in SK-N-MC neuroblastoma cell line as a non-MSC cell model. Hypoxia increased the bindings of both BICD1 and BICD2 to HIF1α in SK-N-MC (Supplementary Fig. S6A). Nuclear HIF1α level in both *BICD1* and *BICD2* siRNAs-cotransfected SK-N-MC with hypoxia is the lowest among the hypoxia-treated experimental groups (Supplementary Fig. S6B). *BICD2* silencing significantly suppressed hypoxia-induced HIF1α nuclear translocation in BICD1 KO SK-N-MC cell line (Supplementary Fig. S7A, B). These results indicate that both BICD1 and BICD2 have a capacity to mediate HIF1α nuclear translocation in SK-N-MCs under hypoxia.

### Hypoxia stimulated the interaction between BICD1 and HIF1α is required for the recruitment of HIF1α to dynein

**Fig. 2:**
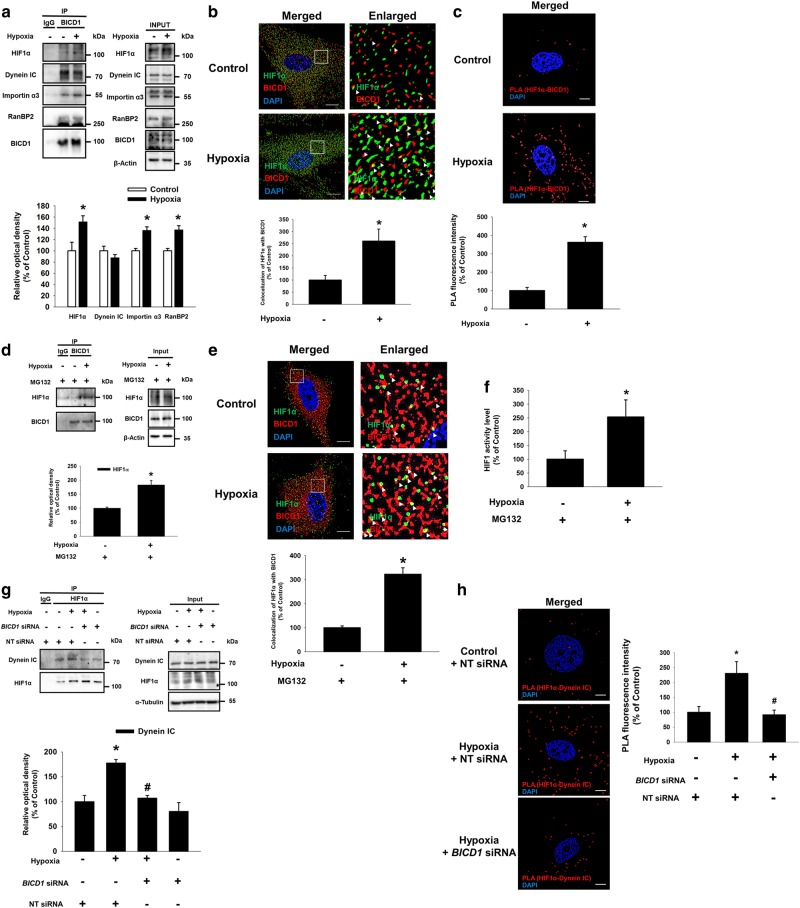
Effect of hypoxia on the interaction between HIF1α and BICD1. **a** Co-immunoprecipitation of HIF1α, Dynein IC, Importin α3, RanBP2 with IgG and BICD1 antibodies were shown in left panel. Total protein expressions in lysate were shown in right panel. *n* = 3. **b** Cells were immunostained with HIF1α and BICD1-specific antibodies. Scale bars are 8 μm (Magnification, × 1,000). White arrow heads indicate co-localization of HIF1α with BICD1. **c** Interaction between HIF1α and BICD1 (HIF1α/BICD1, red) was analyzed by PLA. Scale bars are 8 μm (Magnification, × 1,000). *n* = 6. *p < 0.05 vs. normoxia control. **d–f** Cells were pretreated with MG132 (1 μM) for 30 min prior to hypoxia treatment for 24 h. **d** Co-immunoprecipitation of HIF1α with IgG and BICD1 were shown in left panel. Total protein expressions in lysate were shown in right panel. *n* = 3. **e** Cells were immunostained with HIF1α and BICD1-specific antibodies. White arrow heads indicate co-localization of HIF1α with BICD1 in MG132-pretreated UCB-MSCs. Scale bars are 8 μm (Magnification, × 1,000). *n* = 5. **f** HIF1 activity was measured by dual luciferase reporter assay. *n* = 6. *p < 0.05 vs. normoxia control with MG132 pretreatment. **g**, **h** Cells were transfected with *BICD1* or NT siRNA for 24 h prior to hypoxia treatment for 24 h. **g** Co-immunoprecipitation of Dynein IC with IgG and HIF1α antibodies were shown in left panel. Total protein expressions in lysate were shown in right panel. *n* = 3. **h** Interaction between HIF1α and Dynein IC (HIF1α/Dynein IC, red) was analyzed by PLA. Scale bars are 8 μm (Magnification, × 1,000). Quantitative data are presented as a mean ± S.E.M. All blots and immunofluorescence images are representative. *p < 0.05 vs. normoxia control with NT siRNA transfection, ^#^*p* < 0.05 vs. hypoxia with NT siRNA transfection

Next, we determined the effect of hypoxia on the interaction between HIF1α and BICD1. Our results show that hypoxia stimulated the binding of BICD1 to HIF1α, Importin α3 and RanBP2 (Fig. 2a). Hypoxia significantly increased the co-localization of HIF1α with BICD1 in the cytoplasmic region, which was also shown by the HIF1α/BICD1 proximity ligation assay (PLA) signal compared to the control (Fig. 2b, c). Because the hypoxia-induced HIF1α protein level may contribute to the interaction of HIF1α with BICD1, we did a pretreatment with a proteasome inhibitor MG132 to inhibit the additional induction of the HIF1α protein level by hypoxia. Under the MG132 pretreatment condition, hypoxia stimulated the binding of HIF1α to BICD1 although the total HIF1α protein levels were similar in the UCB-MSCs with or without hypoxia (Fig. 2d). Consistently, the immunocytochemistry and dual luciferase reporter assay data revealed that hypoxia stimulated HIF1 activity as well as the interaction between HIF1α and BICD1 (Fig. 2e, f). In addition, *BICD1* siRNA transfection inhibited the hypoxia-induced binding of HIF1α to Dynein IC (Fig. 2g). The hypoxia-induced PLA signal of HIF1α/Dynein IC was significantly abolished by the *BICD1* siRNA transfection (Fig. 2h). Furthermore, CoCl_2_ treatment induced the binding of HIF1α to Dynein IC and HIF1α nuclear translocation, abolished by *BICD1* silencing (Supplementary Fig. S8A, B).

### Akt-inactivated GSK3β increases the interaction between BICD1 and HIF1α, leading to HIF1α nuclear translocation under hypoxia

**Fig. 3:**
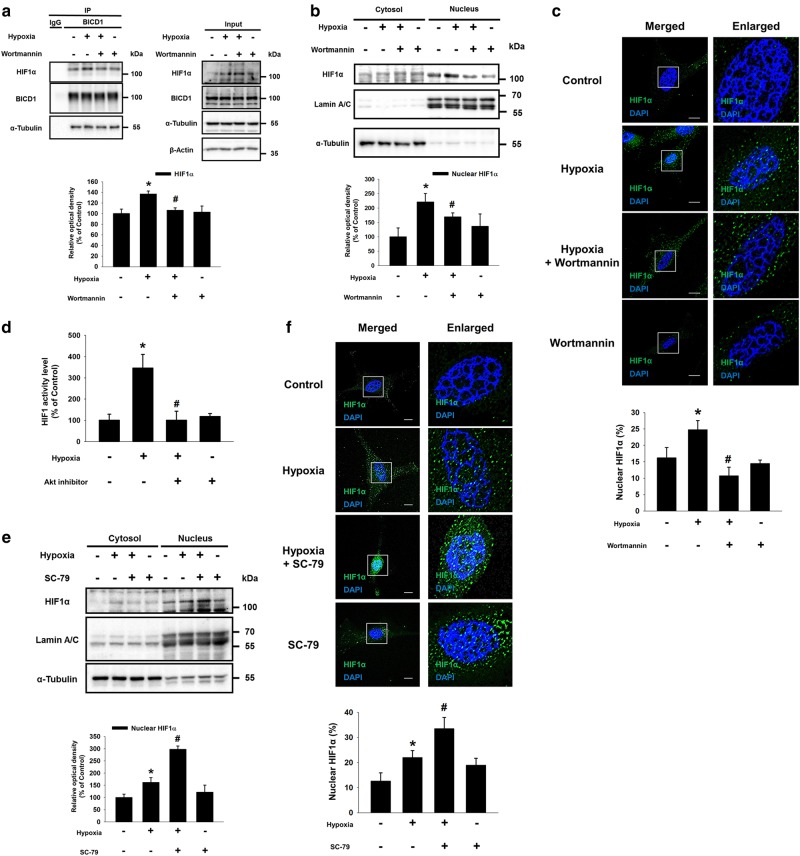
Role of hypoxia-activated Akt in BICD1-mediated HIF1α nuclear translocation. **a–c** The UCB-MSCs were pretreated with wortmannin (1 μM) for 30 min prior to hypoxia treatment for 24 h. **a** Co-immunoprecipitation of HIF1α and α-Tubulin with IgG and BICD1 antibodies were shown in left panel. Total protein expressions in lysate were shown in right panel. *n* = 3. **b** HIF1α, Lamin A/C, and α-Tubulin in cytosolic and nuclear fractionized samples were detected by western blot. *n* = 3. **c** Cells were immunostained with HIF1α-specific antibodies. Scale bars are 8 μm (Magnification, × 1,000). *n* = 4. **d–f** Cells were pretreated with Akt inhibitor (2 μM) or SC-79 (5 μg/mL) for 30 min prior to hypoxia treatment for 24 h. **d** HIF1 activities of UCB-MSCs were measured by dual luciferase reporter assay. *n* = 8. **e** HIF1α, Lamin A/C, and α-Tubulin protein levels in cytosolic and nuclear fractionized samples were analyzed by western blot. *n* = 3. **f** Cells were immunostained with HIF1α-specific antibody. Scale bars are 8 μm (Magnification, × 1,000). *n* = 4. Quantitative data are presented as a mean ± S.E.M. All blots and immunofluorescence images are representative. *p < 0.05 vs. normoxia control, ^#^*p* < 0.05 vs. hypoxia

**Fig. 4:**
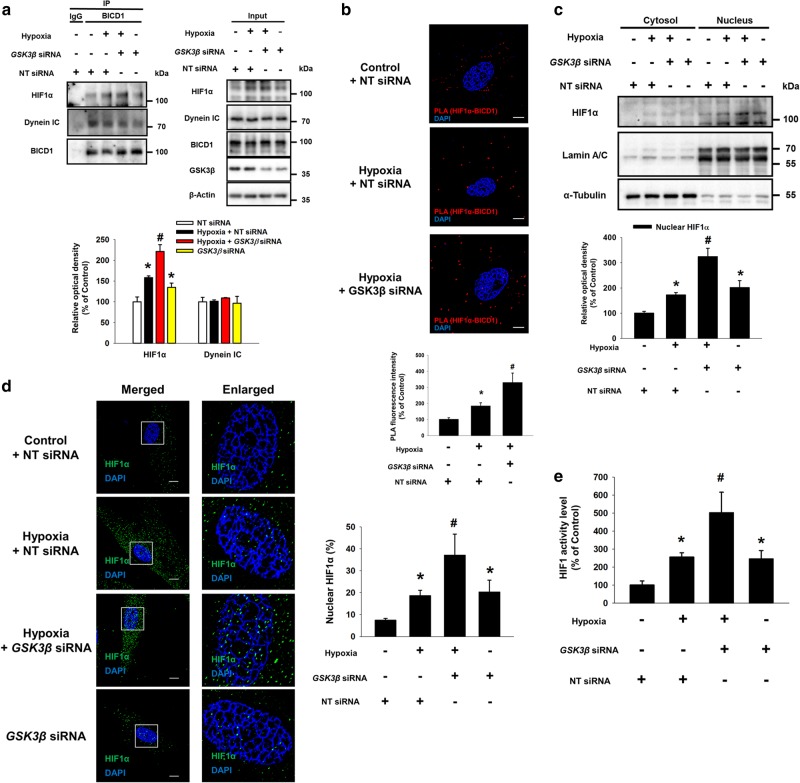
Involvement of Akt/GSK3β pathway in BICD1-mediated HIF1α nuclear translocation. **a–e** The UCB-MSCs were transfected with *GSK3β* or NT siRNA for 24 h prior to hypoxia treatment for 24 h. **a** Co-immunoprecipitation of HIF1α and Dynein IC with IgG and BICD1 antibodies were shown in left panel. Total protein expressions in lysate were shown in right panel. *n* = 3. **b** Interaction between HIF1α and BICD1 (HIF1α/BICD1, red) was analyzed by PLA assay. Scale bars are 8 μm (Magnification, × 1,000). *n* = 5. **c** HIF1α, Lamin A/C, and α-Tubulin in cytosolic and nuclear fractionized samples were detected by western blot. *n* = 4. **d** Cells were immunostained with HIF1α-specific antibody. Scale bars are 8 μm (Magnification, × 1,000). *n* = 4. **e** HIF1 activities in NT or *GSK3β* siRNA-transfected cells were analyzed by dual luciferase reporter assay. *n* = 6. Quantitative data are presented as a mean ± S.E.M. All blots and immunofluorescence images are representative. *p < 0.05 vs. normoxia control with NT siRNA transfection, ^#^*p* < 0.05 vs. hypoxia with NT siRNA transfection

We investigated the mechanism on how BICD1 stimulates hypoxia-induced HIF1α nuclear translocation. Our data revealed that hypoxia inhibited the bindings of BICD1 to Akt and GSK3β (Supplementary Fig. S9). However, no differences were observed in the mRNA expression levels of *BICD1*, *BICD2*, *DYNC1H1*, and *DYNC2H1* between normoxia and hypoxia, suggesting that BICD1 regulation by hypoxia is independent of its protein level (Supplementary Fig. S10). Pretreatment of the PI3K/Akt inhibitor wortmannin suppressed the hypoxia-stimulated binding of BICD1 to HIF1α and the nuclear translocation of HIF1α (Fig. 3a–c). In addition, the dual luciferase reporter assay results also show that Akt inhibitor pretreatment suppressed the hypoxia-increased HIF1 activity (Fig. 3D). Conversely, pretreatment with the Akt activator SC-79 significantly enhanced the hypoxia-stimulated HIF1α nuclear translocation (Fig. 3e, f). Pretreatment with the Akt inhibitor reduced the hypoxia-induced inhibitory phosphorylation of GSK3β at the Ser9 residue, suggesting that hypoxia inactivates GSK3β via Akt (Supplementary Fig. S11). Thus, we further investigated the effect of *GSK3β* silencing on the hypoxia-induced interaction between BICD1 and HIF1α. *GSK3β* siRNA transfection abolished the hypoxia-stimulated binding of BICD1 to HIF1α (Fig. 4a, b). Moreover, an increased HIF1α nuclear translocation and activity in *GSK3β* siRNA-transfected UCB-MSCs with hypoxia compared to the non-targeting (NT) siRNA-transfected UCB-MSCs with hypoxia. And, the *GSK3β* siRNA-transfected UCB-MSCs with normoxia stimulated the HIF1α nuclear translocation and activity compared to the NT siRNA-transfected UCB-MSCs with normoxia (Fig. 4c–e).

### *BICD1* silencing induces the glycolytic switch suppression and mitochondrial ROS accumulation resulting in mitochondrial damage

**Fig. 5:**
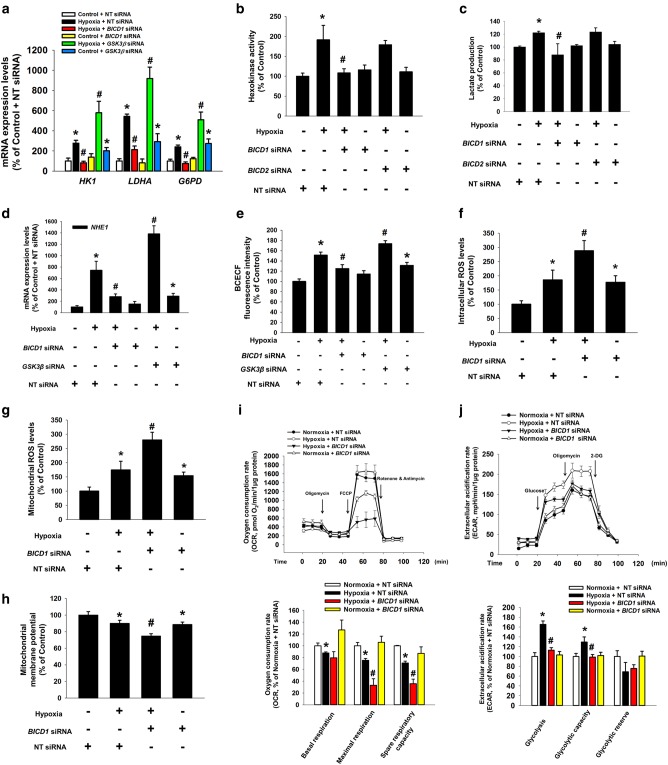
Role of BICD1 or GSK3β in glycolysis and ROS accumulation in UCB-MSCs under hypoxia. (**a–e, i, j**) The UCB-MSCs were transfected *BICD1*, *BICD2*, *GSK3β*, or NT siRNA for 24 h prior to hypoxia treatment for 24 h. **a** mRNA expression levels of *HK1*, *LDHA*, and *G6PD* were analyzed by quantitative real-time PCR. *n* = 5. Gene expression levels were normalized by *18S rRNA* expression level. **b**, **c** Hexokinase activity and lactate production in cells were analyzed by using commercial kit. *n* = 6. **d** mRNA expression level of *NHE1* was analyzed by quantitative real-time PCR. *n* = 5. Gene expression levels were normalized by *18S rRNA* expression level. **e** Intracellular alkalization was measured by BCECF-AM staining. *n* = 8. **f–h** The UCB-MSCs were transfected *BICD1*, *GSK3β*, or NT siRNA for 24 h prior to hypoxia treatment for 48 h. Intracellular ROS, mitochondrial ROS, and mitochondrial membrane potential of cells were measured by luminometer. *n* = 8. **i** Oxygen consumption rate (OCR) changes under mitochondrial stress test in UCB-MSCs under normoxia or hypoxia were measured by using Seahorse XF24 Extracellular Flux analyzer. *n* = 5. Statistics of basal respiration, maximal respiration, and spare respiratory capacity were presented in lower panel. **j** Extracellular acidification rate (ECAR) changes under glycolysis stress test in UCB-MSCs under normoxia or hypoxia were measured by using Seahorse XF24 Extracellular Flux analyzer. *n* = 5. Statistics of glycolysis, glycolytic capacity, and glycolytic reserve were presented in lower panel. Quantitative data are presented as a mean ± S.E.M. *p < 0.05 vs. normoxia control with NT siRNA transfection, ^#^*p* < 0.05 vs. hypoxia with NT siRNA transfection

We investigated the effect of BICD1 regulation on glycolysis and intracellular ROS accumulation in UCB-MSCs. Our data revealed that hypoxia increased the mRNA expression levels of HIF1-targeted glycolysis enzymes including *HK1*, *LDHA*, and *G6PD* and other HIF1 target genes including EPO and BNIP3 were inhibited by *BICD1* siRNA transfection; however, they were enhanced by *GSK3β* siRNA transfection (Fig. 5a and Supplementary Fig. S12). Hypoxia-stimulated hexokinase activity and lactate production were abolished by transfection of *BICD1* siRNA but not by *BICD2* siRNA (Fig. 5b, c). Because recent studies have shown that chronic hypoxia-induced intracellular alkalization is favorable for glycolysis [37, 38], we investigated the role of BICD1 in hypoxia-induced Na^+^/H^+^ exchanger isoform 1 (NHE1) expression and intracellular alkalization in UCB-MSCs. *NHE1* mRNA expression and BCECF-AM fluorescence intensity were abolished by *BICD1* siRNA transfection; however, they were further increased by *GSK3β* siRNA transfection (Fig. 5d, e). Total and mitochondrial ROS levels in NT siRNA-transfected UCB-MSCs with hypoxia were higher than those in *BICD1* siRNA-transfected UCB-MSCs with hypoxia (Fig. 5f, g). *BICD1* siRNA transfection decreased the mitochondrial membrane potential of the UCB-MSCs under hypoxia (Fig. 5h). OCR data showed that hypoxia decreased basal respiration, maximal respiration and spare respiratory capacity. *BICD1* silencing further decreased maximal respiration and spare respiratory capacity (Fig. 5i). In addition, ECAR data showed that hypoxia increased glycolysis and glycolytic capacity, suppressed by *BICD1* silencing (Fig. 5j). These data indicates that BICD1 plays an important role in hypoxia-stimulated glycolytic reprogramming of UCB-MSCs.

### Hypoxia-regulated BICD1 is important for survival of UCB-MSCs

**Fig. 6:**
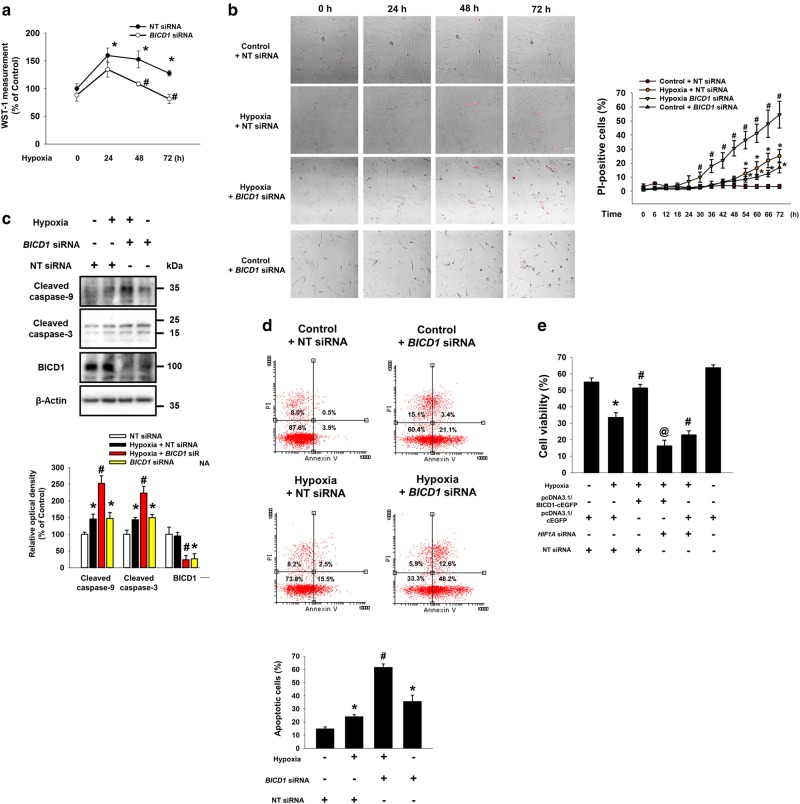
Role of BICD1 in UCB-MSCs survival under hypoxia. **a**, **b** The UCB-MSCs were transfected with *BICD1* or NT siRNA for 24 h prior to hypoxia treatment. **a** Cells were exposed to various durations of hypoxia (0–72 h). Cell viability of UCB-MSCs was measured by WST-1 cell viability assay. *n* = 8. **b** Cells were incubated in normoxia or hypoxia conditions for 72 h. Representative images of experimental groups at 0, 24, 48, and 72 h of normoxia or hypoxia incubation are presented. Red-marked cells in representative images indicate PI-positive cells. *n* = 4. Scale bars are 50 μm (Magnification, × 200). **c** *BICD1* or NT siRNA-transfected cells were incubated in hypoxia for 24 h. Cleaved caspase-9, cleaved caspase-3, and β-Actin protein expressions were detected by western blot analysis. *n* = 4. **d** Cells were transfected with *BICD1* or NT siRNA for 24 h prior to hypoxia treatment for 48 h. The percentages of apoptotic cells were analyzed by Annexin V/PI analysis, measured by flowcytometer. Annexin V-positive cells were considered as apoptotic cells. *n* = 4. Quantitative data are presented as a mean ± S.E.M. All blot images are representative. *p < 0.05 vs. normoxia control with NT siRNA transfection, ^#^*p* < 0.05 vs. hypoxia with NT siRNA transfection. **e** The UCB-MSCs were transfected with pcDNA3.1/BICD1-cEGFP vector, pcDNA3.1/cEGFP vector, *HIF1A* siRNA or NT siRNA for 24 h prior to hypoxia treatment for 72 h. Cell viability data are presented as a mean ± S.E.M. *n* = 5. *p *<* 0.05 vs. normoxia control with pcDNA3.1/cEGFP vector and NT siRNA cotransfection, ^#^*p* *<* 0.05 vs. hypoxia control with pcDNA3.1/cEGFP vector and NT siRNA cotransfection, @*p* *<* 0.05 vs. hypoxia control with pcDNA3.1/BICD1-cEGFP vector and NT siRNA cotransfection

Furthermore, we investigated the effect of *BICD1* or *BICD2* siRNA transfection on the proliferation and survival of UCB-MSCs under hypoxia. Cell proliferation and viability assay data revealed that the proliferation and viability of *BICD1* siRNA-transfected UCB-MSCs were significantly lower than that of the NT siRNA-transfected UCB-MSCs during 48–72 h of hypoxia (Fig. 6a). Live-cell imaging results show that PI-positive cells in *BICD1* siRNA-transfected UCB-MSCs under hypoxia was significantly higher than that in NT siRNA-transfected UCB-MSCs under hypoxia during 30–72 h (Fig. 6b). Moreover, both PI-positive cells in NT siRNA-transfected UCB-MSCs under hypoxia and that in *BICD1* siRNA-transfected UCB-MSCs under normoxia are higher than that in NT siRNA-transfected UCB-MSCs under normoxia during 54–72 h (Fig. 6b). *BICD1* siRNA transfection increased the expressions of cleaved caspase-9 and -3 in UCB-MSCs with normoxia and hypoxia (Fig. 6c). The apoptosis of the *BICD1* siRNA-transfected UCB-MSCs with hypoxia was significantly higher than that of the NT siRNA-transfected UCB-MSCs with hypoxia (Fig. 6d). In addition, BICD1 overexpression increased survival rate of UCB-MSCs under hypoxia, completely abolished by *HIF1α* silencing (Fig. 6e).

### BICD1 regulation by *GSK3β* silencing enhances the regenerative potential of hypoxia-pretreated UCB-MSCs

**Fig. 7:**
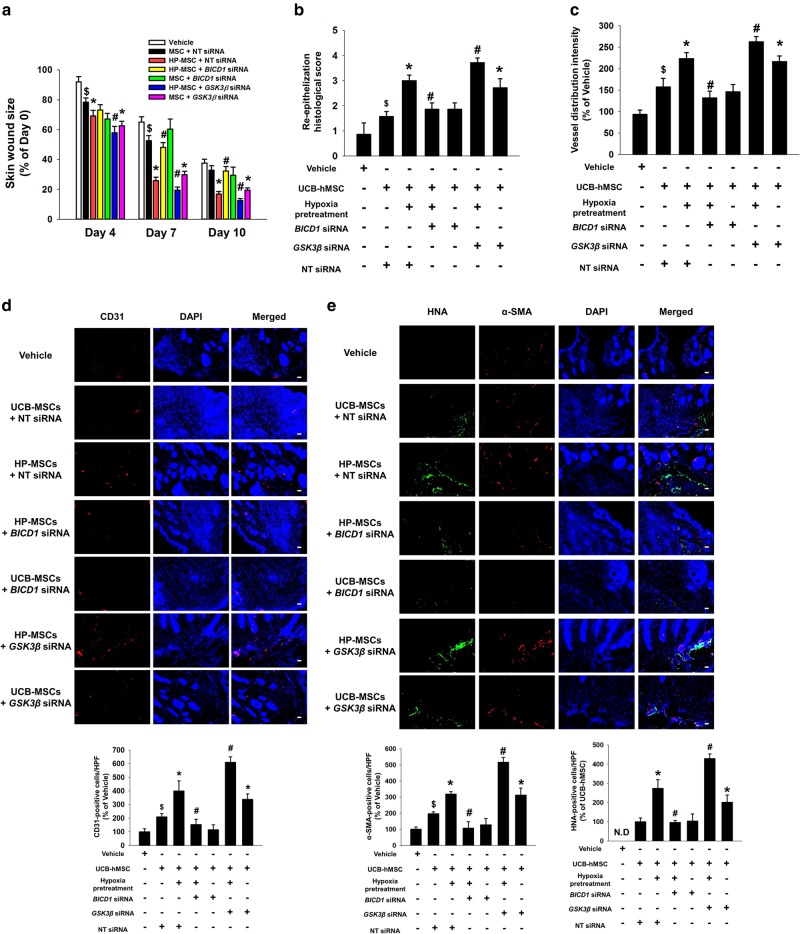
Effect of BICD1 regulation on skin wound healing capacity of UCB-MSC transplantation. **a–e** Mouse skin wound surgery with vehicle or UCB-MSC transplantation was conducted as described in Materials and methods. HP-MSC indicates mice group given hypoxia-pretreated UCB-MSCs with NT siRNA. **a** Skin wound size was compared with post-injection day 0. *n* = 12. **b** Tissue samples at post-injection day 10 were stained with hematoxylin and eosin. Scale bars are 200 μm (Magnification, × 40). *n* = 7. **c** Vessel distribution was quantified with ImageJ software. *n* = 10. **d**, **e** Tissue slides were immunostained with CD31, HNA, and α-SMA-specific antibodies. The percentages of CD31, HNA, and α-SMA-positive cells in DAPI-positive were analyzed by using Metamorph software. Scale bars are 100 μm (Magnification, × 100). *n* = 4. Quantitative data are presented as a mean ± S.E.M. All gross and immunofluorescence images are representative. ^$^*p* < 0.05 vs. vehicle group, *p < 0.05 vs. MSC group with NT siRNA, ^#^*p* < 0.05 vs. HP-MSC with NT siRNA. N.D. indicates not detected

At 7 and 10 days after the skin wound surgery, the wound area in mice given the hypoxia-pretreated UCB-MSCs with *BICD1* siRNA was greater than that in mice given the hypoxia-pretreated UCB-MSCs with NT siRNA. This wound healing effect of the hypoxia-pretreated UCB-MSCs or UCB-MSCs with NT siRNA was further enhanced by *GSK3β* siRNA transfection (Fig. 7a and Supplementary Fig. S13A). In the histological assessment with hematoxylin & eosin-stained skin samples at 10 days after the skin wound surgery, the reepithelization histological score of the hypoxia-pretreated UCB-MSCs was higher than that of the hypoxia-pretreated UCB-MSCs with *BICD1* siRNA and lower than that of the hypoxia-pretreated UCB-MSCs with *GSK3β* siRNA. The histological score of the hypoxia-pretreated UCB-MSCs with *GSK3β* siRNA was the highest among all the experimental groups (Fig. 7b and Supplementary Fig. S13B). In addition, we assessed whether *BICD1* or *GSK3β* siRNA transfection regulates neovascularization induced by MSC transplantation in the wound healing process. As shown in Fig. 7c, d and Supplementary Fig. S13C, the vessel distribution intensity and the number of pan-endothelial marker CD31-positive cells in the wound site of mice given the hypoxia-pretreated UCB-MSCs with NT siRNA were significantly higher than that given the hypoxia-pretreated UCB-MSCs with *BICD1* siRNA and lower than that of the hypoxia-pretreated UCB-MSCs with *GSK3β* siRNA. The amount of myofibroblast marker α-smooth muscle actin (α-SMA) and human nuclear antigen (HNA), a marker for transplanted UCB-MSCs, -positive cells at the wound site had a similar pattern in mice given the hypoxia-pretreated UCB-MSCs with NT, *BICD1* or *GSK3β* siRNA (Fig. 7e).

## Discussion

This study highlights the regulatory role of BICD1 in dynein-mediated HIF1α nuclear translocation and the mechanism inducing the interaction between BICD1 and HIF1α under hypoxia. Given there has been no report presenting BICD1 as a regulator of HIF1α nuclear translocation under hypoxia, we are the first to report that dynein-mediated HIF1α nuclear transport is dependent on BICD1. Consistent with our results, it has been reported that the overexpression of full length BICD increased the cargo transport ability of dynein [15]. Moreover, the addition of the N-terminal of BICD2 stabilized the complex of dynein and dynactin, and then, the dynein complex became highly processive [39, 40]. Taken together, these findings support our data indicating that BICD1 is a regulatory factor inducing the HIF1α nuclear transport capacity of dynein. Here, we also provide ample evidence that hypoxia-stimulates direct binding of BICD1 to HIF1α, and this interaction between BICD1 and HIF1α is independent of the HIF1α protein level. Although the HIF1α-specific binding domain of BICD1 has not been reported, the coiled-coil region 3 (CC3) in the C-terminal of BICD1 is considered a cargo binding motif for several proteins such as Rab6, dynactin, and RanBP2 [41–43]. These reports suggest that the CC3 of BICD1 could be a possible binding site for HIF1α. In addition, we found that hypoxia stimulated the interaction of BICD1 with Importin α3 and RanBP2 as well as HIF1α. Importin α3 was reported as a major isotype which interacts with the C-terminal nuclear localization signal (NLS) region of HIF1α, important for its nuclear localization [44]. Importin α bound to NLS forms a heterodimer complex with Importin β, leading to the docking to RanBP2 for nuclear import [45–47]. A previous researcher showed that RanBP2 interacts with BICD2 but not BICD1 [18]. However, other researchers demonstrated that the positively charged basic 1 region in CC3 of BICD1 has a crucial role in RanBP2 recruitment, consistent with our data [42, 43]. These reports indicate that the binding affinities of BICD1 and BICD2 against RanBP2 appeared to be cell type-specific. Although the role of RanBP2-induced SUMOylation of cargo including HIF1α in its nuclear import has been controversial, the N-terminal leucine rich domain and putative coiled-coil domain of RanBP2 are required for Importin-mediated nuclear import of cargo [20, 48]. Therefore, these findings present BICD1 as a direct interacting partner of HIF1α for its nuclear translocation in UCB-MSCs. Previous studies reported the physiological roles of BICD1 and BICD2 in neuronal cells. BICD1 is required for dendrite branch formation, and BICD2 mutation impairs dynein-mediated trafficking and neurite growth [49, 50]. Based upon results with SK-N-MC cell models, we suggested that BICD2-mediated HIF1α nuclear translocation in SK-N-MCs may be related to structural similarity between BICDs. However, contributions of BICDs to HIF1α nuclear translocation appear cell type-specific.

Because our data revealed that hypoxia did not affect the expression level of BICD1, we hypothesized that hypoxia stimulates the interaction between BICD1 and HIF1α through BICD1 activation for HIF1α nuclear translocation. Moreover, we demonstrated that the Akt/GSK3β pathway is a key pathway regulating the BICD1-mediated HIF1α nuclear translocation in UCB-MSCs under hypoxia. It has been well documented that hypoxia-inactivated GSK3β is induced by Akt phosphorylation [51, 52]. Therefore, these findings suggest that Akt activation or *GSK3β* silencing is an efficient strategy for enhanced BICD1-mediated HIF1α nuclear translocation. A previous researcher reported that GSK3β stimulated phosphorylation of the CC3 domain of BICD1 at the Ser585 and Thr597 residues, which induces the anchoring of BICD1 to γ-Tubulin in the centrosome [53]. The centrosomal localization of BICD1 and the interaction between BICD1 and dynein were abolished by a loss-of-function mutation of BICD1, suggesting that GSK3β decreases the cargo nuclear transport ability of BICD1 [53]. Remarkably, a previous study reported that *GSK3β* silencing abolished the interaction between BICD1 and the centrosomal protein ninein [53]; however, our data revealed that it stimulated the interaction between BICD1 and HIF1α. These findings suggest that the effect of BICD1 phosphorylation by GSK3β on the binding affinity to cargo proteins may vary depending on the protein type. Taken together, we present GSK3β as a regulator for the BICD1-mediated HIF1α nuclear translocation in UCB-MSCs under hypoxia.

Although there has been no report determining the role of BICD in glycolysis metabolism and ROS regulation under hypoxia, our data show that BICD1-silenced UCB-MSCs exhibited decrease of mitochondrial respiration capacity, impairment of glycolytic reprogramming and dysregulation of the mitochondrial ROS level. The changes of maximal mitochondrial respiration and mitochondrial respiratory capacity are associated with metabolic substrate availability, mitochondrial content and integrity [54–56]. A previous report showed that spare respiratory capacity regulated by mitochondrial complex II contributes to cell survival under hypoxia [54]. Indeed, our data showed that *BICD1* silencing promoted apoptosis of UCB-MSCs under hypoxia. Glycolysis-induced cells exhibit an increased extracellular acidification rate, lactate production, and hexokinase activity [57]. In addition, cellular alkalization during hypoxia is mainly stimulated by the HIF1-induced NHE1 and monocarboxylate transporter MCT1 expressions which evacuate H^+^ and lactate, respectively [58–60]. Given that our results revealed that *BICD1* silencing inhibited HIF1-targeted glycolysis enzymes and NHE1-induced alkalization, it is implied that the suppression of glycolytic reprogramming by *BICD1* silencing is induced by the suppression of HIF1α nuclear translocation. Glycolytic reprogramming during hypoxia adaptation prevents excessive mitochondrial ROS accumulation due to the slowing of electron transport and the reduction of NADH oxidation [61]. Many studies have reported that metabolic reprogramming during hypoxia adaptation reduces the cytotoxic ROS level, closely associated with cell survival [62]. Indeed, our previous studies showed that aberrant control of the mitochondrial ROS level is a major factor resulting in apoptosis of UCB-MSCs during long term exposure to hypoxia [33]. Thus, we suggest that the BICD1-mediated metabolic switch to glycolysis prevents cytotoxic ROS accumulation, leading to the anti-apoptosis of UCB-MSCs under hypoxia. In addition to HIF1α, we studied the effect of *BICD1* silencing on another ROS regulator Nrf2 expression and nuclear translocation in UCB-MSCs under hypoxia. However, we found that both hypoxia and *BICD1* silencing did not affect the Nrf2 expression level and its nuclear translocation (Supplementary Figs. S14A, B). Although many previous investigators reported that hypoxia stimulates Nrf2 expression and activity [63, 64], other investigators also reported that hypoxia and CoCl_2_ treatment does not change the Nrf2 expression and nuclear translocation [65, 66]. Especially, a previous investigator reported that CoCl_2_ treatment does not affect the nuclear Nrf2 expression level in UCB-MSCs [65]. Furthermore, the present study showed that the silencing of *GSK3β* as a BICD1 regulator enhanced glycolysis metabolism adapted to hypoxia. Many researchers have reported the regulatory effect of the Akt/GSK3β pathway on HIF1-mediated glycolysis. Akt activation using platelet-derived growth factor induced glycolysis through HIF1 activation [31]. Moreover, the Akt/mTOR inhibitor Jolkinolide B suppressed glycolysis through the inhibition of HK2 expression [67]. We also provided in vivo evidence that the transplanted cell survival and skin wound healing capacity of UCB-MSCs with hypoxia pretreatment is dependent on BICD1 regulation by GSK3β. It has been reported that glycolysis adapted by hypoxia is essential for improving the survival and therapeutic efficacy of hypoxia-preconditioned MSC therapy in ischemic tissue [68]. Consistent with our results, the inhibition of the Akt/GSK3β signaling by lithium or lysophosphatidic acid enhanced the therapeutic efficacy of transplanted MSCs [69, 70]. Overall, we suggest that BICD1 activation regulated by GSK3β is a promising strategy for improving the transplantation efficacy and regenerative potential of hypoxia-pretreated UCB-MSCs.

**Fig. 8:**
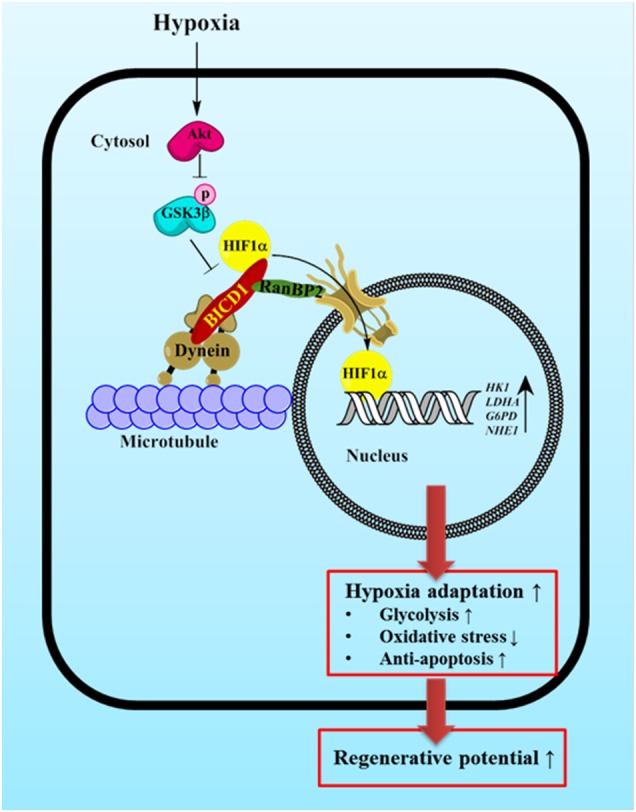
The schematic model for mechanism of BICD1-mediated HIF1α nuclear translocation in UCB-MSCs under hypoxia. Hypoxia induces GSK3β phosphorylation at Ser9 residue via Akt activation. Silencing of *GSK3β* induces the interaction between BICD1 and HIF1α. BICD1 regulation by Akt activation or *GSK3β* silencing stimulates HIF1α nuclear translocation. BICD1-mediated HIF1α nuclear translocation is critical for hypoxia adaptation, enhances regenerative potential of UCB-MSCs transplantation

In conclusion, we demonstrated that hypoxia stimulates the interaction between BICD1 and HIF1α resulting in BICD1-mediated HIF1α nuclear translocation via the Akt/GSK3β pathway. Activation of BICD1 by GSK3β inhibition enhances the hypoxia adaptation to glycolysis and the survival of UCB-MSCs, leading to the increased regenerative potential of transplanted UCB-MSCs with hypoxia pretreatment (Fig. 8). Our investigation is the first identification of BICD1 as a regulator of HIF1α nuclear translocation leading to hypoxia adaptation. Although further investigation into the binding sequence of HIF1α interacting with BICD1 is required to find other BICD1-regulated transcription factors, present study provides new insight into the HIF1α-specific therapeutic strategy for MSC-based therapy.

## Electronic supplementary material

Supplementary figure legends

Supplementary figure S1

Supplementary figure S2

Supplementary figure S3

Supplementary figure S4

Supplementary figure S5

Supplementary figure S6

Supplementary figure S7

Supplementary figure S8

Supplementary figure S9

Supplementary figure S10

Supplementary figure S11

Supplementary figure S12

Supplementary figure S13

Supplementary figure S14

Supplementary tables

## References

[1] Kim JW, Tchernyshyov I, Semenza GL, Dang CV (2006). HIF-1-mediated expression of pyruvate dehydrogenase kinase: a metabolic switch required for cellular adaptation to hypoxia. Cell Metab.

[2] Zhang CC, Sadek HA (2014). Hypoxia and metabolic properties of hematopoietic stem cells. Antioxid Redox Signal.

[3] Chen J, Kang JG, Keyvanfar K, Young NS, Hwang PM (2016). Long-term adaptation to hypoxia preserves hematopoietic stem cell function. Exp Hematol.

[4] Choudhry H, Harris AL (2018). Advances in hypoxia-inducible factor biology. Cell Metab.

[5] Ito K, Suda T (2014). Metabolic requirements for the maintenance of self-renewing stem cells. Nat Rev Mol Cell Biol.

[6] Saito S, Lin YC, Tsai MH, Lin CS, Murayama Y, Sato R (2015). Emerging roles of hypoxia-inducible factors and reactive oxygen species in cancer and pluripotent stem cells. Kaohsiung J Med Sci.

[7] Koyasu S, Kobayashi M, Goto Y, Hiraoka M, Harada H (2018). Regulatory mechanisms of hypoxia-inducible factor 1 activity: Two decades of knowledge. Cancer Sci.

[8] Jiang X, Zhang D, Zhang H, Huang Y, Teng M (2015). Role of Ran-regulated nuclear-cytoplasmic trafficking of pVHL in the regulation of microtubular stability-mediated HIF-1α in hypoxic cardiomyocytes. Sci Rep.

[9] Guo H, Zheng H, Wu J, Ma HP, Yu J, Yiliyaer M (2017). The key role of microtubules in hypoxia preconditioning-induced nuclear translocation of HIF-1α in rat cardiomyocytes. PeerJ.

[10] Teng M, Dang YM, Zhang JP, Zhang Q, Fang YD, Ren J (2010). Microtubular stability affects cardiomyocyte glycolysis by HIF-1α expression and endonuclear aggregation during early stages of hypoxia. Am J Physiol Heart Circ Physiol.

[11] Carbonaro M, Escuin D, O’Brate A, Thadani-Mulero M, Giannakakou P (2012). Microtubules regulate hypoxia-inducible factor-1α protein trafficking and activity: implications for taxane therapy. J Biol Chem.

[12] Hoogenraad CC, Wulf P, Schiefermeier N, Stepanova T, Galjart N, Small JV (2003). Bicaudal D induces selective dynein-mediated microtubule minus end-directed transport. EMBO J.

[13] Hoogenraad CC, Akhmanova A, Howell SA, Dortland BR, De Zeeuw CI, Willemsen R (2001). Mammalian golgi-associated bicaudal-D2 functions in the dynein-dynactin pathway by interacting with these complexes. EMBO J.

[14] Dharan A, Opp S, Abdel-Rahim O, Keceli SK, Imam S, Diaz-Griffero F (2017). Bicaudal D2 facilitates the cytoplasmic trafficking and nuclear import of HIV-1 genomes during infection. Proc Natl Acad Sci USA.

[15] Matanis T, Akhmanova A, Wulf P, Del Nery E, Weide T, Stepanova T (2002). Bicaudal-D regulates COPI-independent Golgi-ER transport by recruiting the dynein-dynactin motor complex. Nat Cell Biol.

[16] Liu Y, Salter HK, Holding AN, Johnson CM, Stephens E, Lukavsky PJ (2013). Bicaudal-D uses a parallel, homodimeric coiled coil with heterotypic registry to coordinate recruitment of cargos to dynein. Genes Dev.

[17] Budzinska M, Wicher KB, Terenzio M (2017). Neuronal roles of the bicaudal D family of motor adaptors. Vitam Horm.

[18] Splinter D, Tanenbaum ME, Lindqvist A, Jaarsma D, Flotho A, Yu KL (2010). Bicaudal D2, dynein, and kinesin-1 associate with nuclear pore complexes and regulate centrosome and nuclear positioning during mitotic entry. PLoS Biol.

[19] Goldberg MW (2017). Nuclear pore complex tethers to the cytoskeleton. Semin Cell Dev Biol.

[20] Walde S, Thakar K, Hutten S, Spillner C, Nath A, Rothbauer U (2012). The nucleoporin Nup358/RanBP2 promotes nuclear import in a cargo- and transport receptor-specific manner. Traffic.

[21] Qiao C, Xu W, Zhu W, Hu J, Qian H, Yin Q (2008). Human mesenchymal stem cells isolated from the umbilical cord. Cell Biol Int.

[22] Wang M, Yang Y, Yang D, Luo F, Liang W, Guo S (2009). The immunomodulatory activity of human umbilical cord blood-derived mesenchymal stem cells in vitro. Immunology.

[23] Yubo M, Yanyan L, Li L, Tao S, Bo L, Lin C (2017). Clinical efficacy and safety of mesenchymal stem cell transplantation for osteoarthritis treatment: A meta-analysis. PLoS ONE.

[24] Volkman R, Offen D (2017). Concise review: Mesenchymal stem cells in neurodegenerative diseases. Stem Cells.

[25] Palomaki S, Pietila M, Laitinen S, Pesala J, Sormunen R, Lehenkari P (2013). HIF-1α is upregulated in human mesenchymal stem cells. Stem Cells.

[26] Bader AM, Klose K, Bieback K, Korinth D, Schneider M, Seifert M (2015). Hypoxic preconditioning increases survival and pro-angiogenic capacity of human cord blood mesenchymal stromal cells in vitro. PLoS ONE.

[27] Lee Jun, Yoon Yeo, Lee Sang (2017). Hypoxic Preconditioning Promotes the Bioactivities of Mesenchymal Stem Cells via the HIF-1α-GRP78-Akt Axis. International Journal of Molecular Sciences.

[28] Liu YY, Chiang CH, Hung SC, Chian CF, Tsai CL, Chen WC (2017). Hypoxia-preconditioned mesenchymal stem cells ameliorate ischemia/reperfusion-induced lung injury. PLoS ONE.

[29] Abkhezr M, Keramati AR, Ostad SN, Davoodi J, Ghahremani MH (2010). The time course of Akt and ERK activation on XIAP expression in HEK 293 cell line. Mol Biol Rep.

[30] Harada H, Itasaka S, Kizaka-Kondoh S, Shibuya K, Morinibu A, Shinomiya K (2009). The Akt/mTOR pathway assures the synthesis of HIF-1α protein in a glucose- and reoxygenation-dependent manner in irradiated tumors. J Biol Chem.

[31] Lambert CM, Roy M, Robitaille GA, Richard DE, Bonnet S (2010). HIF-1 inhibition decreases systemic vascular remodelling diseases by promoting apoptosis through a hexokinase 2-dependent mechanism. Cardiovasc Res.

[32] Chachami G, Hatziefthimiou A, Liakos P, Ioannou MG, Koukoulis GK, Bonanou S (2007). Exposure of differentiated airway smooth muscle cells to serum stimulates both induction of hypoxia-inducible factor-1α and airway responsiveness to ACh. Am J Physiol Lung Cell Mol Physiol.

[33] Lee HJ, Jung YH, Choi GE, Ko SH, Lee SJ, Lee SH (2017). BNIP3 induction by hypoxia stimulates FASN-dependent free fatty acid production enhancing therapeutic potential of umbilical cord blood-derived human mesenchymal stem cells. Redox Biol.

[34] Onphachanh Xaykham, Lee Hyun Jik, Lim Jae Ryong, Jung Young Hyun, Kim Jun Sung, Chae Chang Woo, Lee Sei-Jung, Gabr Amr Ahmed, Han Ho Jae (2017). Enhancement of high glucose-induced PINK1 expression by melatonin stimulates neuronal cell survival: Involvement of MT2 /Akt/NF-κB pathway. Journal of Pineal Research.

[35] Gustafsson N, Culley S, Ashdown G, Owen DM, Pereira PM, Henriques R (2016). Fast live-cell conventional fluorophore nanoscopy with ImageJ through super-resolution radial fluctuations. Nat Commun.

[36] Chan DA, Sutphin PD, Denko NC, Giaccia AJ (2002). Role of prolyl hydroxylation in oncogenically stabilized hypoxia-inducible factor-1α. J Biol Chem.

[37] Yao H, Zhao H, Wang J, Haddad GG (2018). Intracellular pH regulation in iPSCs-derived astrocytes from subjects with chronic mountain sickness. Neuroscience.

[38] Fan Q, Yang L, Zhang X, Ma Y, Li Y, Dong L (2018). Autophagy promotes metastasis and glycolysis by upregulating MCT1 expression and Wnt/β-catenin signaling pathway activation in hepatocellular carcinoma cells. J Exp Clin Cancer Res.

[39] Trokter M, Mucke N, Surrey T (2012). Reconstitution of the human cytoplasmic dynein complex. Proc Natl Acad Sci USA.

[40] Cianfrocco MA, Leschziner AE (2014). Traffic control: adaptor proteins guide dynein-cargo takeoff. EMBO J.

[41] Kardon JR, Vale RD (2009). Regulators of the cytoplasmic dynein motor. Nat Rev Mol Cell Biol.

[42] Terawaki S, Yoshikane A, Higuchi Y, Wakamatsu K (2015). Structural basis for cargo binding and autoinhibition of Bicaudal-D1 by a parallel coiled-coil with homotypic registry. Biochem Biophys Res Commun.

[43] Terawaki S, Ootsuka H, Higuchi Y, Wakamatsu K (2014). Crystallographic characterization of the C-terminal coiled-coil region of mouse Bicaudal-D1 (BICD1). Acta Crystallogr F Struct Biol Commun.

[44] Depping R, Steinhoff A, Schindler SG, Friedrich B, Fagerlund R, Metzen E (2008). Nuclear translocation of hypoxia-inducible factors (HIFs): involvement of the classical importin α/β pathway. Biochim Biophys Acta.

[45] Miyamoto Y, Yamada K, Yoneda Y (2016). Importin α: a key molecule in nuclear transport and non-transport functions. J Biochem.

[46] Hutten S, Flotho A, Melchior F, Kehlenbach RH (2008). The Nup358-RanGAP complex is required for efficient importin α/β-dependent nuclear import. Mol Biol Cell.

[47] Hamada M, Haeger A, Jeganathan KB, van Ree JH, Malureanu L, Walde S (2011). Ran-dependent docking of importin-β to RanBP2/Nup358 filaments is essential for protein import and cell viability. J Cell Biol.

[48] Melchior F, Schergaut M, Pichler A (2003). SUMO: ligases, isopeptidases and nuclear pores. Trends Biochem Sci.

[49] Aguirre-Chen C, Bulow HE, Kaprielian ZC (2011). elegans bicd-1, homolog of the Drosophila dynein accessory factor Bicaudal D, regulates the branching of PVD sensory neuron dendrites. Development.

[50] Oates EC, Rossor AM, Hafezparast M, Gonzalez M, Speziani F, MacArthur DG (2013). Mutations in BICD2 cause dominant congenital spinal muscular atrophy and hereditary spastic paraplegia. Am J Hum Genet.

[51] Beitner-Johnson D, Rust RT, Hsieh TC, Millhorn DE (2001). Hypoxia activates Akt and induces phosphorylation of GSK-3 in PC12 cells. Cell Signal.

[52] Deguchi JO, Yamazaki H, Aikawa E, Aikawa M (2009). Chronic hypoxia activates the Akt and β-catenin pathways in human macrophages. Arterioscler, Thromb Vasc Biol.

[53] Fumoto K, Hoogenraad CC, Kikuchi A (2006). GSK-3β-regulated interaction of BICD with dynein is involved in microtubule anchorage at centrosome. EMBO J.

[54] Pfleger J, He M, Abdellatif M (2015). Mitochondrial complex II is a source of the reserve respiratory capacity that is regulated by metabolic sensors and promotes cell survival. Cell Death Dis.

[55] Fisher-Wellman KH, Weber TM, Cathey BL, Brophy PM, Gilliam LA, Kane CL (2014). Mitochondrial respiratory capacity and content are normal in young insulin-resistant obese humans. Diabetes.

[56] Schottl T, Kappler L, Fromme T, Klingenspor M (2015). Limited OXPHOS capacity in white adipocytes is a hallmark of obesity in laboratory mice irrespective of the glucose tolerance status. Mol Metab.

[57] Fang Y, Liu Z, Chen Z, Xu X, Xiao M, Yu Y (2017). Smad5 acts as an intracellular pH messenger and maintains bioenergetic homeostasis. Cell Res.

[58] Shimoda LA, Fallon M, Pisarcik S, Wang J, Semenza GL (2006). HIF-1 regulates hypoxic induction of NHE1 expression and alkalinization of intracellular pH in pulmonary arterial myocytes. Am J Physiol: Lung Cell Mol Physiol.

[59] Dayan F, Mazure NM, Brahimi-Horn MC, Pouyssegur J (2008). A dialogue between the hypoxia-inducible factor and the tumor microenvironment. Cancer Microenviron.

[60] Ambrosetti D, Dufies M, Dadone B, Durand M, Borchiellini D, Amiel J (2018). The two glycolytic markers GLUT1 and MCT1 correlate with tumor grade and survival in clear-cell renal cell carcinoma. PLoS ONE.

[61] Eales KL, Hollinshead KE, Tennant DA (2016). Hypoxia and metabolic adaptation of cancer cells. Oncogenesis.

[62] Kim J, Kim J, Bae JS (2016). ROS homeostasis and metabolism: a critical liaison for cancer therapy. Exp Mol Med.

[63] Kolamunne RT, Dias IH, Vernallis AB, Grant MM, Griffiths HR (2013). Nrf2 activation supports cell survival during hypoxia and hypoxia/reoxygenation in cardiomyoblasts; the roles of reactive oxygen and nitrogen species. Redox Biol.

[64] Zhao R, Feng J, He G (2016). Hypoxia increases Nrf2-induced HO-1 expression via the PI3K/Akt pathway. Front Biosci (Landmark Ed).

[65] Yuan Z, Zhang J, Huang Y, Zhang Y, Liu W, Wang G (2017). NRF2 overexpression in mesenchymal stem cells induces stem-cell marker expression and enhances osteoblastic differentiation. Biochem Biophys Res Commun.

[66] Kim TH, Hur EG, Kang SJ, Kim JA, Thapa D, Lee YM (2011). NRF2 blockade suppresses colon tumor angiogenesis by inhibiting hypoxia-induced activation of HIF-1α. Cancer Res.

[67] Gao X, Han H (2018). Jolkinolide B inhibits glycolysis by downregulating hexokinase 2 expression through inactivating the Akt/mTOR pathway in non-small cell lung cancer cells. J Cell Biochem.

[68] Zhu H, Sun A, Zou Y, Ge J (2014). Inducible metabolic adaptation promotes mesenchymal stem cell therapy for ischemia: a hypoxia-induced and glycogen-based energy prestorage strategy. Arterioscler Thromb Vasc Biol.

[69] Chuang DM, Wang Z, Chiu CT (2011). GSK-3 as a target for lithium-induced neuroprotection against excitotoxicity in neuronal cultures and animal models of ischemic stroke. Front Mol Neurosci.

[70] Ryu JM, Han HJ (2015). Autotaxin-LPA axis regulates hMSC migration by adherent junction disruption and cytoskeletal rearrangement via LPAR1/3-dependent PKC/GSK3β/β-catenin and PKC/Rho GTPase pathways. Stem Cells.

