# Supplementary material for: BICD1 mediates HIF1α nuclear translocation in mesenchymal stem cells during hypoxia adaptation
Source: Cell Death Differ. 2018 Nov 21;26(9):1716–34. doi: 10.1038/s41418-018-0241-1 (PMC6748134; doi:10.1038/s41418-018-0241-1)
Supplement: Supplementary file 1 — Supplementary figure legends [file 41418_2018_241_MOESM1_ESM.docx]

**Supplementary figure legends**

**Figure S1. Effect of microtubule depolymerization and dynein inhibition on HIF1α nuclear translocation under hypoxia.** (A-C) The UCB-MSCs were pretreated with nocodazole (200 nM) or ciliobrevin D (20 μM) for 30 min prior to hypoxia incubation for 24 h. (A, B) HIF1α, Lamin A/C and α-Tubulin in cytosolic and nuclear fractionized samples were detected by western blot. (C) Cells were immunostained with HIF1α-specific antibody. Scale bars are 8 μm (Magnification, × 1,000). *n*=4. Quantitative data are presented as a mean ± S.E.M. Blot images are representative. **p* < 0.05 versus normoxia control, #*p* < 0.05 versus hypoxia.

**Figure S2. Effect of dynein inhibition on apoptosis of UCB-MSCs under hypoxia.** (A, B) The UCB-MSCs were pretreated with ciliobrevin D (20 μM) for 30 min prior to hypoxia treatment for 48 h. (A) Cleaved caspase-9 and β-Actin were detected by western blot analysis. *n*=3. (B) The percentage of apoptotic cells was analyzed by Annexin V/PI analysis, measured by flowcytometer. Annexin V-positive cells were considered as apoptotic cells. *n*=4. Quantitative data are presented as a mean ± S.E.M. Blot images are representative. **p* < 0.05 versus normoxia control, #*p* < 0.05 versus hypoxia.

**Figure S3. Role of dynein in the interaction of HIF1, BICD1 and BICD2 with α-Tubulin.** The UCB-MSCs were pretreated ciliobrevin D (20 μM) for 30 min prior to hypoxia treatment for 24 h. Co-immunoprecipitation of HIF1α, BICD1, BICD2 with IgG and α-Tubulin antibodies were shown in left panel. Total protein expressions in lysate were shown in right panel. *n*=3. Quantitative data are presented as a mean ± S.E.M. Blot images are representative. **p* < 0.05 versus normoxia control, #*p* < 0.05 versus hypoxia.

**Figure S4. Overexpression of BICD1 by plasmid transfection.** The UCB-MSCs were transfected with plasmid vector transfection for 24 h prior to hypoxia treatment 24 h. BICD1 and β-Actin expressions were analyzed by western blot. *n*=3. Quantitative data are presented as a mean ± S.E.M. Blot images are representative. **p* < 0.05 versus normoxia control with pcDNA3.1/BICD1-cEGFP plasmid transfection.

**Figure S5. Effects of BICD1 silencing and overexpression on prolyl hydroxylation of HIF1α.** (A) NT or BICD1 siRNA-transfected UCB-MSCs were pretreated with MG132 (1 μM) for 30 min prior to hypoxia treatment for 24 h. Hyp402 HIF1α, Hyp564 HIF1α, HIF1α, BICD1 and β-Actin were detected by western blot. n=4. All blot images are representative. Relative optical density data are shown as a mean ± S.E.M. *p<0.05 versus normoxia control with NT siRNA and MG132. (B) BICD1 overexpression vector (pcDNA3.1/BICD1-cEGFP) or non-targeting vector (pcDNA3.1/cEGFP)-transfected UCB-MSCs pretreated with MG132 (1 μM) for 30 min prior to hypoxia treatment for 24 h. Hyp402 HIF1α, Hyp564 HIF1α, HIF1α, BICD1 and β-Actin expressions were detected by western blot. n=4. All blot images are representative. Relative optical density data are shown as a mean ± S.E.M. *p<0.05 versus normoxia control with pcDNA3.1/cEGFP vector transfection.

**Figure S6.** **Effect of BICD1 and BICD2 silencing on HIF1α nuclear translocation in SK-N-MCs under hypoxia.** (A) The SK-N-MCs were treated with hypoxia for 24 h. HIF1α-interacted proteins were immunoprecipitated, and blotted with BICD1, BICD2 and HIF1α antibodies. Cell lysates as an input were blotted with BICD1, BICD2, HIF1α and β-Actin antibodies. All blot images are representative. Relative optical density data are shown as a mean ± S.E.M. *n=3*. **p* < 0.05 versus normoxia control. (B) The SK-N-MCs were transfected with *BICD1*, *BICD2* or NT siRNA for 24 h prior to hypoxia for 24h. HIF1α, Lamin A/C and α-Tubulin in cytosolic and nuclear fractionized samples were detected by western blot. *n*=3. All blot images are representative. Relative optical density data are shown as a mean ± S.E.M. **p* < 0.05 versus normoxia control with NT siRNA transfection, #*p* < 0.05 versus hypoxia control with NT siRNA transfection.

**Figure S7. Effect of BICD2 silencing on HIF1α nuclear translocation in BICD1 knock out SK-N-MC under hypoxia.** (A) BICD1 and β-Actin in BICD1 knock out and control SK-N-MCs were detected by western blot. (B) BICD1 knock out (BICD1 KO) SK-N-MCs were transfected with *BICD2* or NT siRNA for 24 h prior to hypoxia for 24h. HIF1α, Lamin A/C and α-Tubulin in cytosolic and nuclear fractionized samples were detected by western blot. *n*=3. All blot images are representative. Relative optical density data are shown as a mean ± S.E.M. **p* < 0.05 versus normoxia control with NT siRNA transfection, #*p* < 0.05 versus hypoxia control with NT siRNA transfection.

**Figure S8. Effect of BICD1 silencing on CoCl_2_-stimulated HIF1α nuclear translocation.** (A, B) The UCB-MSCs were transfected with *BICD1* or NT siRNAs for 24 h prior to CoCl_2_ (100 μM) pretreatment for 24 h. (A) HIF1α-interacted proteins were immunoprecipitated, and blotted with Dynein IC and HIF1α antibodies. Cell lysates as an input were blotted with Dynein IC, HIF1α and α-Tubulin. (B) HIF1α, Lamin A/C and α-Tubulin in cytosolic and nuclear fractionized samples were detected by western blot. *n*=4. All blot images are representative. Relative optical density data are shown as a mean ± S.E.M. *n=3*. **p* < 0.05 versus normoxia control with NT siRNA, #*p* < 0.05 versus CoCl_2_ with NT siRNA.

**Figure S9. Effect of hypoxia on the interaction of Akt and GSK3β with BICD1.** Co-immunoprecipitation of Akt and GSK3β with BICD1 and IgG antibodies were shown in left panel. Total protein expressions in lysate were shown in right panel. *n*=3. Quantitative data are presented as a mean ± S.E.M. Blot images are representative. **p* < 0.05 versus normoxia control.

**Figure S10. Effect of hypoxia on BICD and dynein expressions.** The UCB-MSCs were treated with hypoxia for 24 h. mRNA expressions of *BICD1*, *BICD2*, *DYNCH1* and *DYNC2H1* were analyzed by quantitative real-time PCR. Gene expression levels were normalized by *ACTB* mRNA expression level. *n*=4. N.S indicates not significant.

**Figure S11. Role of Akt in hypoxia-induced GSK3β phosphorylation.** The UCB-MSCs were pretreated with Akt inhibitor (2 μM) for 30 min prior to hypoxia treatment for 24 h. Phosphorylation of GSK3β at Ser9 residue was analyzed by western blot. *n*=4. Quantitative data are presented as a mean ± S.E.M. All blot images are representative. **p* < 0.05 versus normoxia control, #*p* < 0.05 versus hypoxia.

**Figure S12. Effect of BICD1 or GSK3β silencing on *EPO* and *BNIP3* mRNA expressions.** The UCB-MSCs were transfected with *BICD1*, *GSK3β* or NT siRNA for 24 h prior to hypoxia treatment for 24 h. mRNA expression levels of *EPO* and *BNIP3* were analyzed by quantitative real-time PCR. *n*=5. Gene expression levels were normalized by *18S rRNA* expression level. Quantitative data are presented as a mean ± S.E.M. **p* < 0.05 versus normoxia control with NT siRNA transfection, #*p* < 0.05 versus hypoxia with NT siRNA transfection.

**Figure S13. Representative images of skin wound, H&E staining and blood vessel distribution around wound site.** (A-C) Mouse skin wound procedure was described in Materials and Methods. (A) Representative gross images (left panel) acquired at post-injection day 0, 4, 7 and 10. (B) Representative histological gross images were shown. C, crust; GT, granulated tissue; D, dermis. (C) Representative images of blood vessel distribution around wound site were shown.

**Figure S14. Effect of BICD1 silencing on Nrf2 expression and nuclear translocation in UCB-MSCs under hypoxia.** (A, B) The UCB-MSCs were transfected with *BICD1* or NT siRNA for 24 h prior to hypoxia treatment for 24 h. (A) Nrf 2 and β-Actin expression were detected by western blot. *n*=3. (B) Nrf2, Lamin A/C and α-Tubulin in cytosolic and nuclear fractionized samples were detected by western blot. All blot images are representative. Relative optical density data are shown as a mean ± S.E.M. N.S. indicates not significant.
