# Supplementary figures and images for: BICD1 mediates HIF1α nuclear translocation in mesenchymal stem cells during hypoxia adaptation
Source: Cell Death Differ. 2018 Nov 21;26(9):1716–34. doi: 10.1038/s41418-018-0241-1 (PMC6748134; doi:10.1038/s41418-018-0241-1)

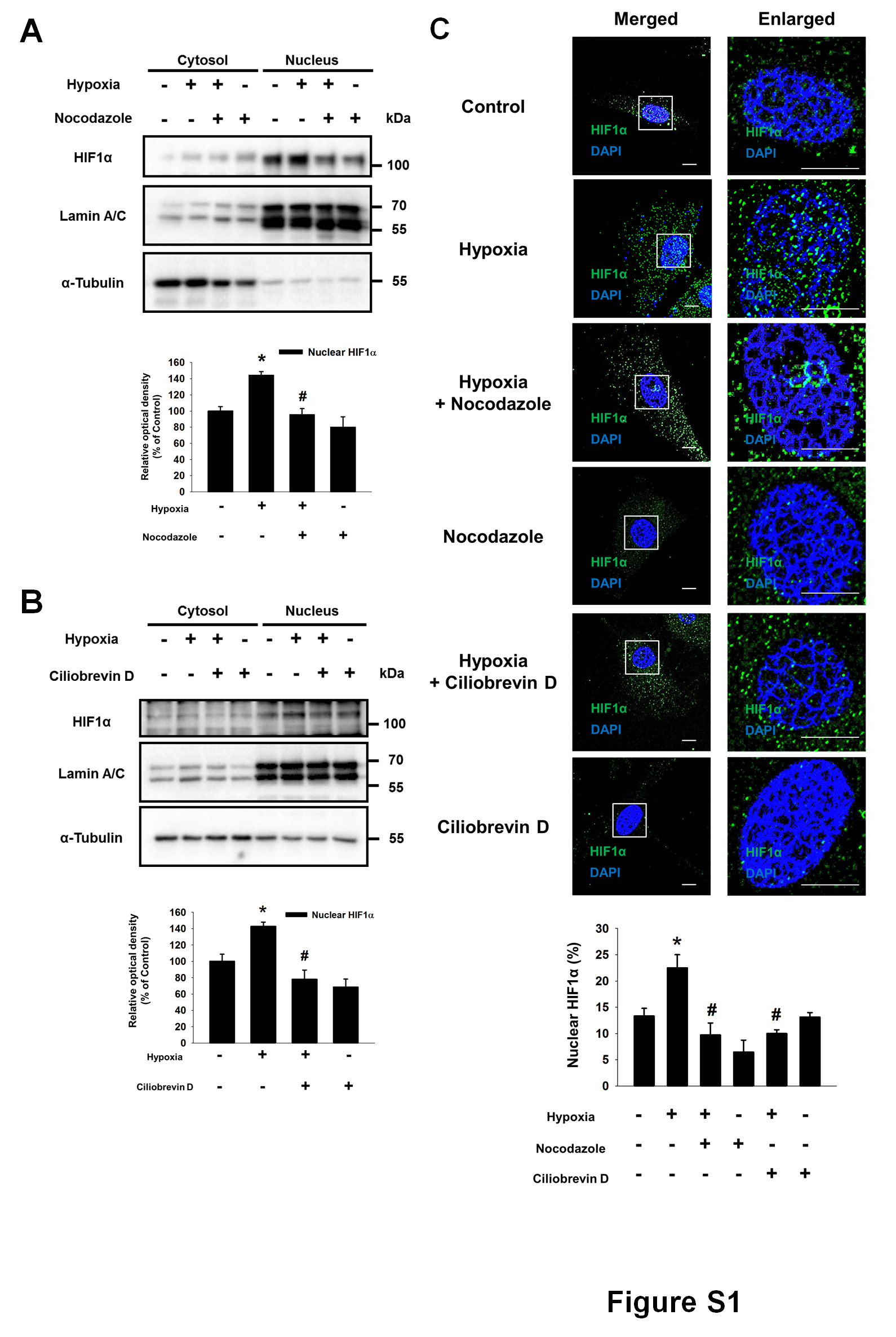

Supplement: Supplementary file 2 — Supplementary figure S1 [file 41418_2018_241_MOESM2_ESM.tif]

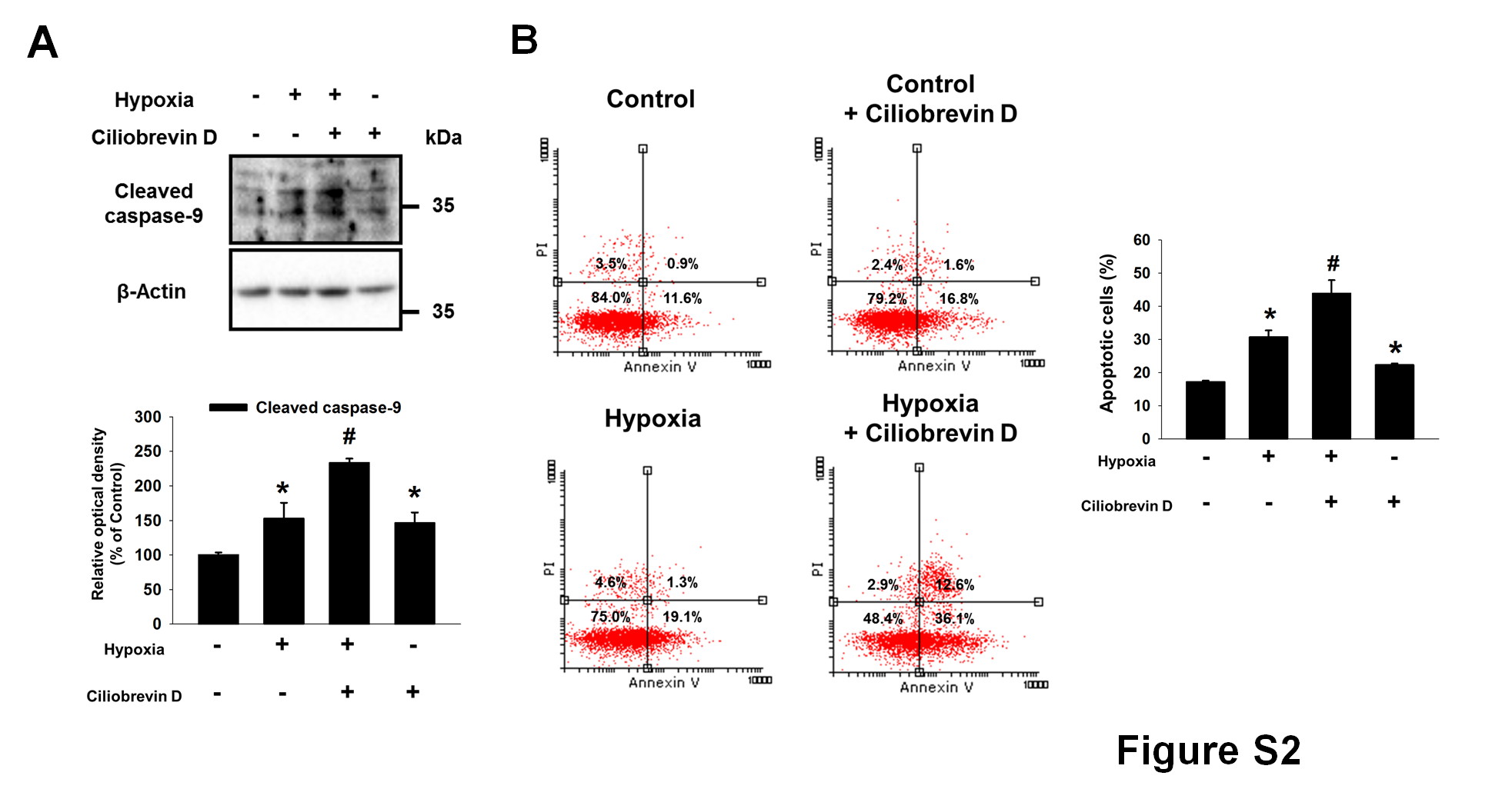

Supplement: Supplementary file 3 — Supplementary figure S2 [file 41418_2018_241_MOESM3_ESM.tif]

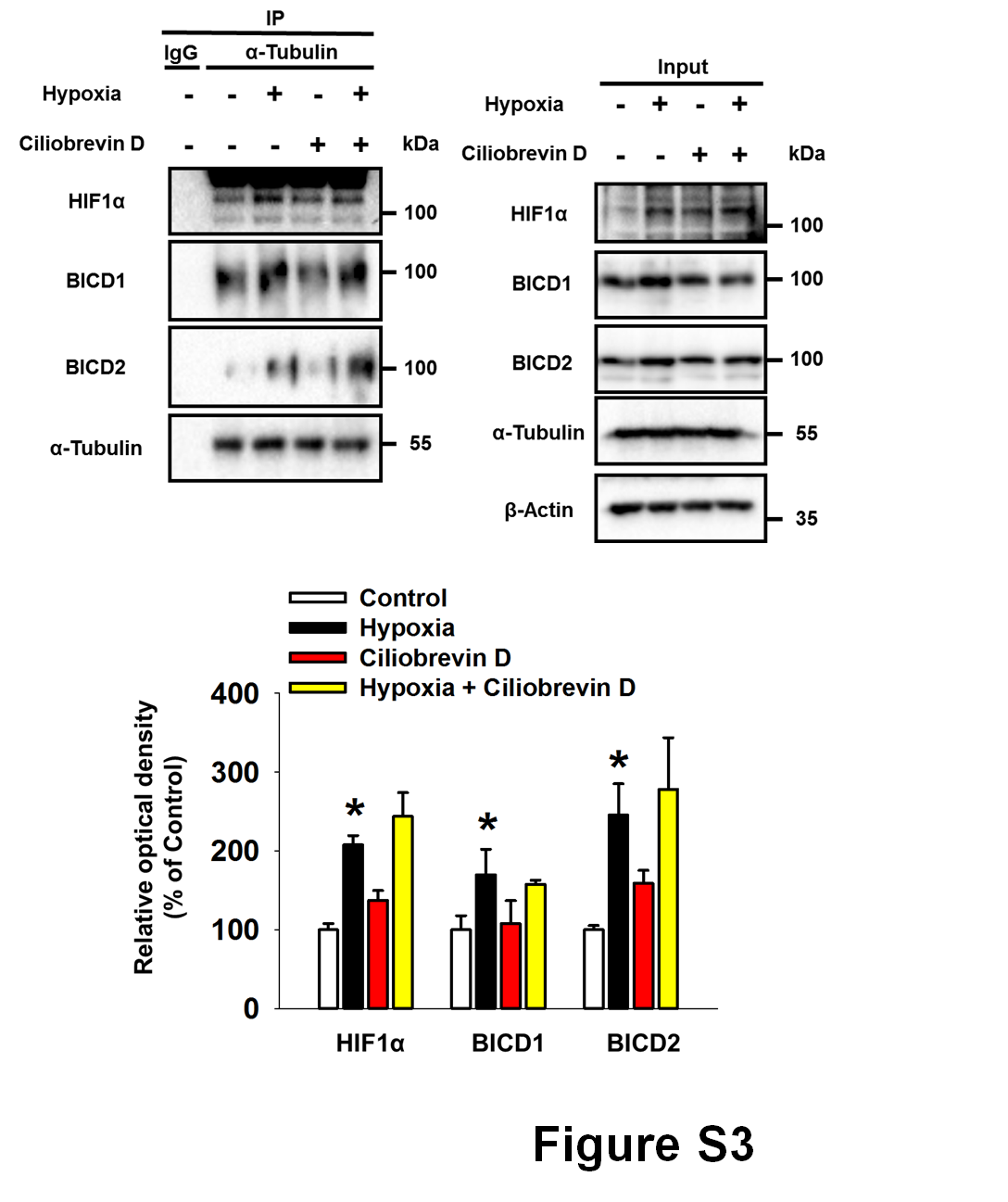

Supplement: Supplementary file 4 — Supplementary figure S3 [file 41418_2018_241_MOESM4_ESM.tif]

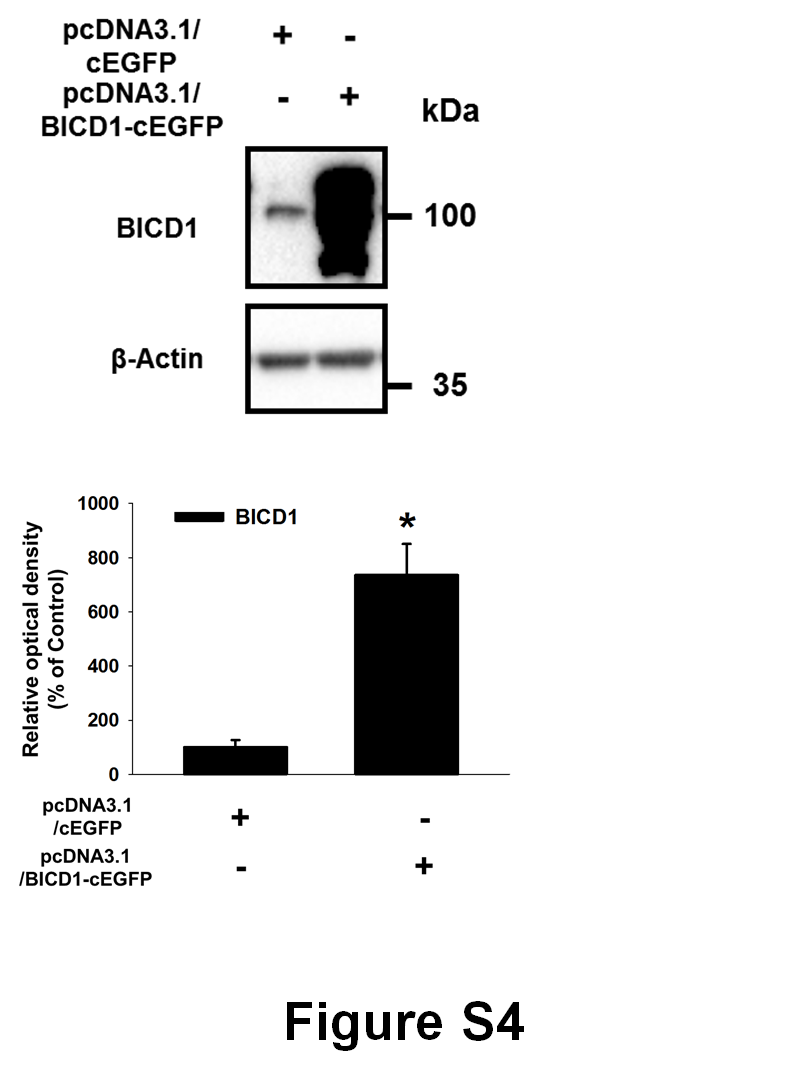

Supplement: Supplementary file 5 — Supplementary figure S4 [file 41418_2018_241_MOESM5_ESM.tif]

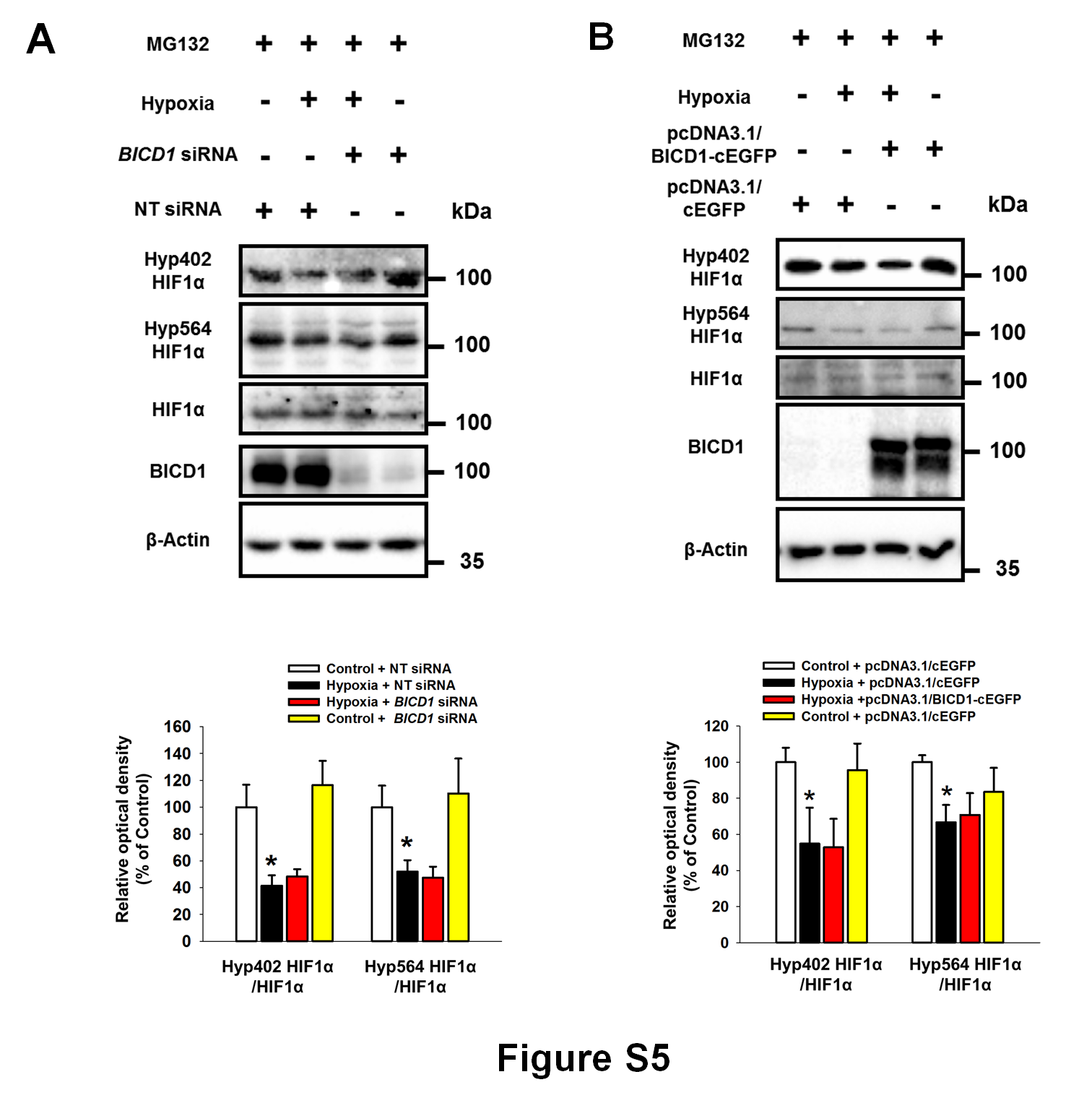

Supplement: Supplementary file 6 — Supplementary figure S5 [file 41418_2018_241_MOESM6_ESM.tif]

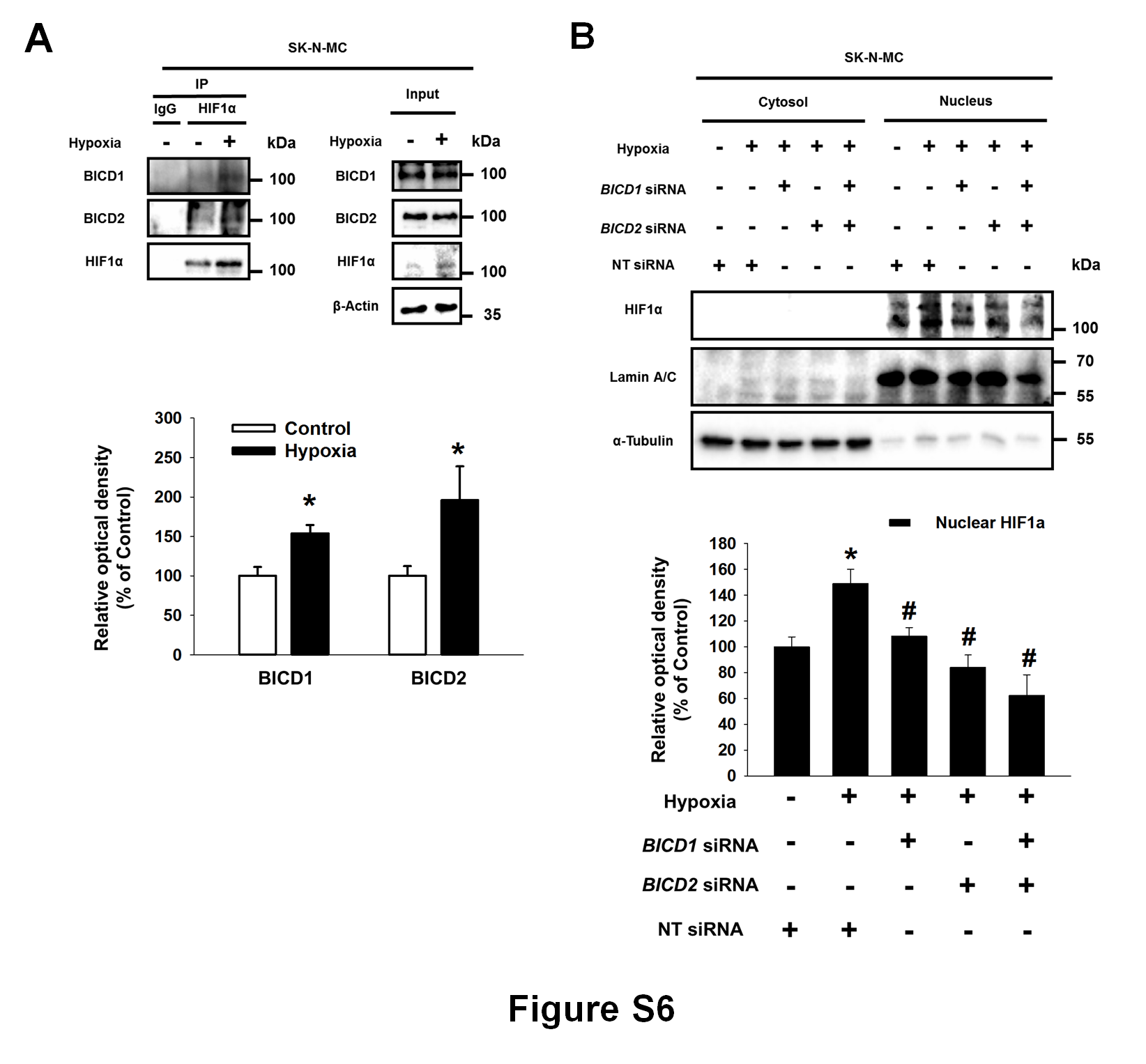

Supplement: Supplementary file 7 — Supplementary figure S6 [file 41418_2018_241_MOESM7_ESM.tif]

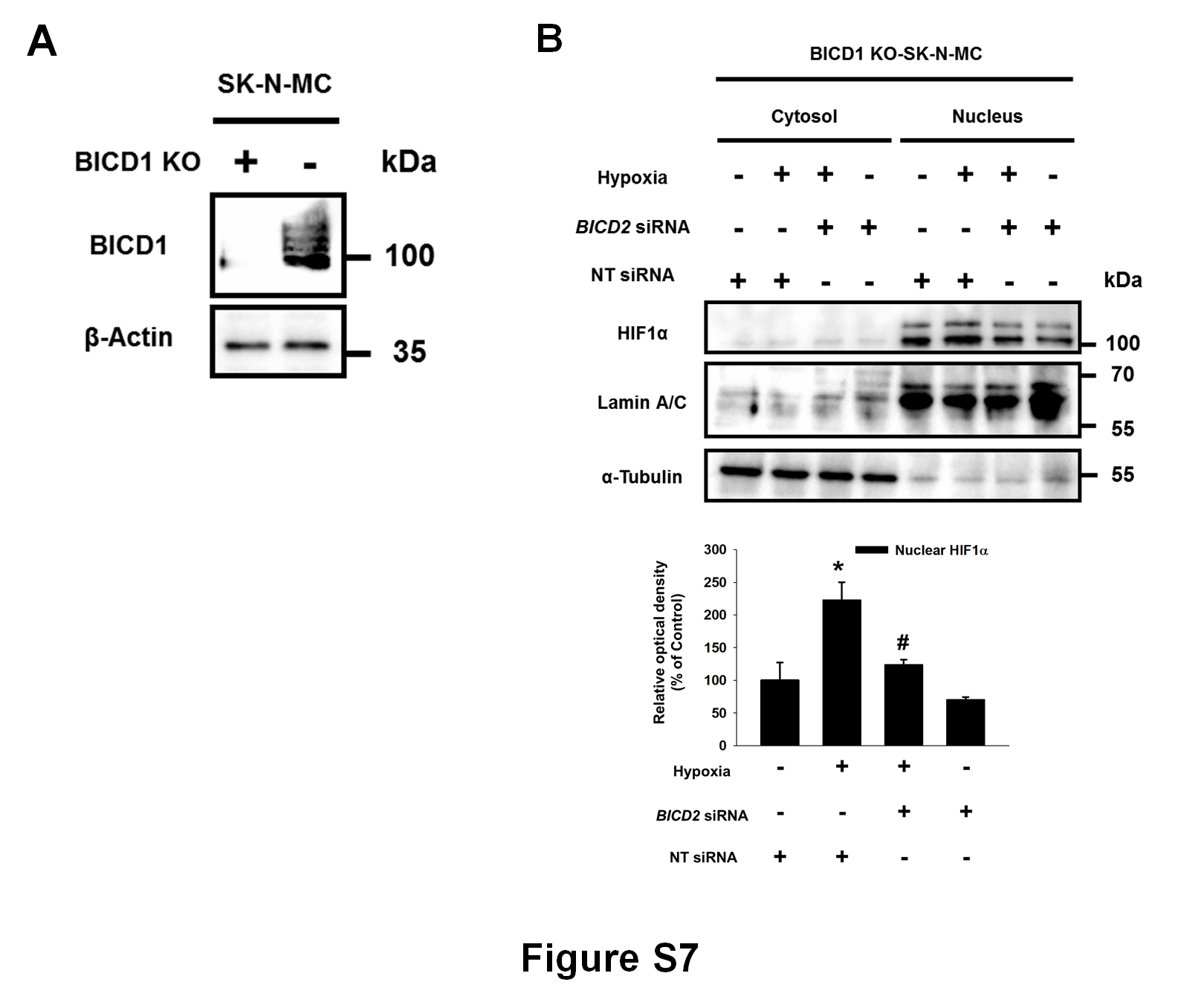

Supplement: Supplementary file 8 — Supplementary figure S7 [file 41418_2018_241_MOESM8_ESM.tif]

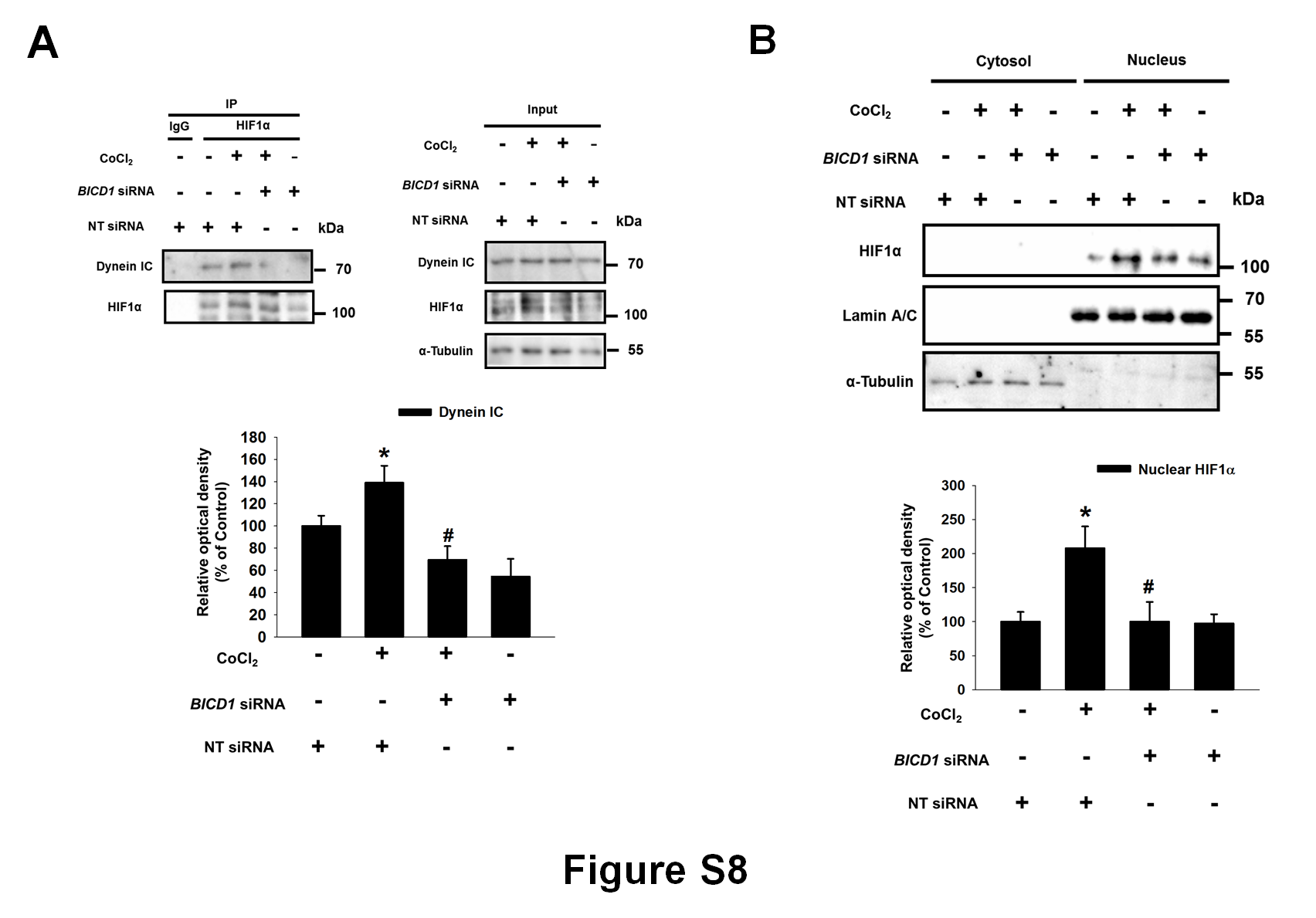

Supplement: Supplementary file 9 — Supplementary figure S8 [file 41418_2018_241_MOESM9_ESM.tif]

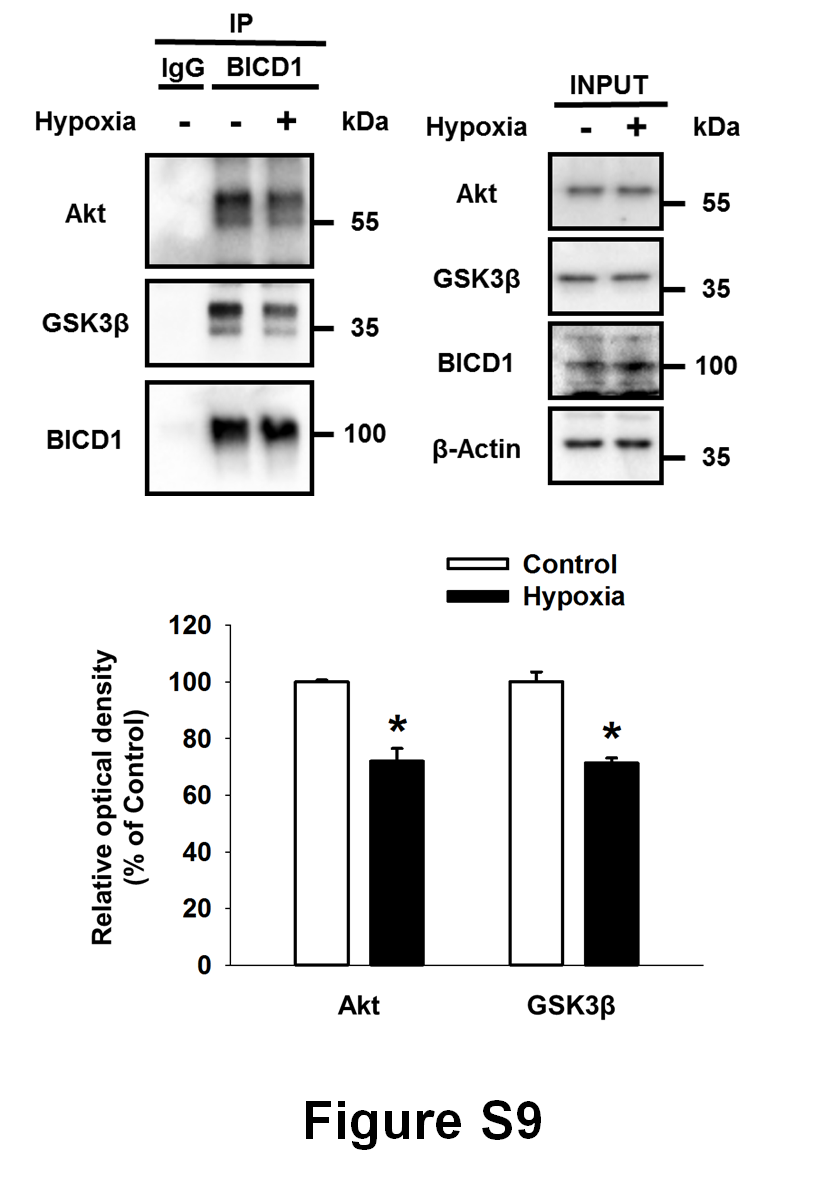

Supplement: Supplementary file 10 — Supplementary figure S9 [file 41418_2018_241_MOESM10_ESM.tif]

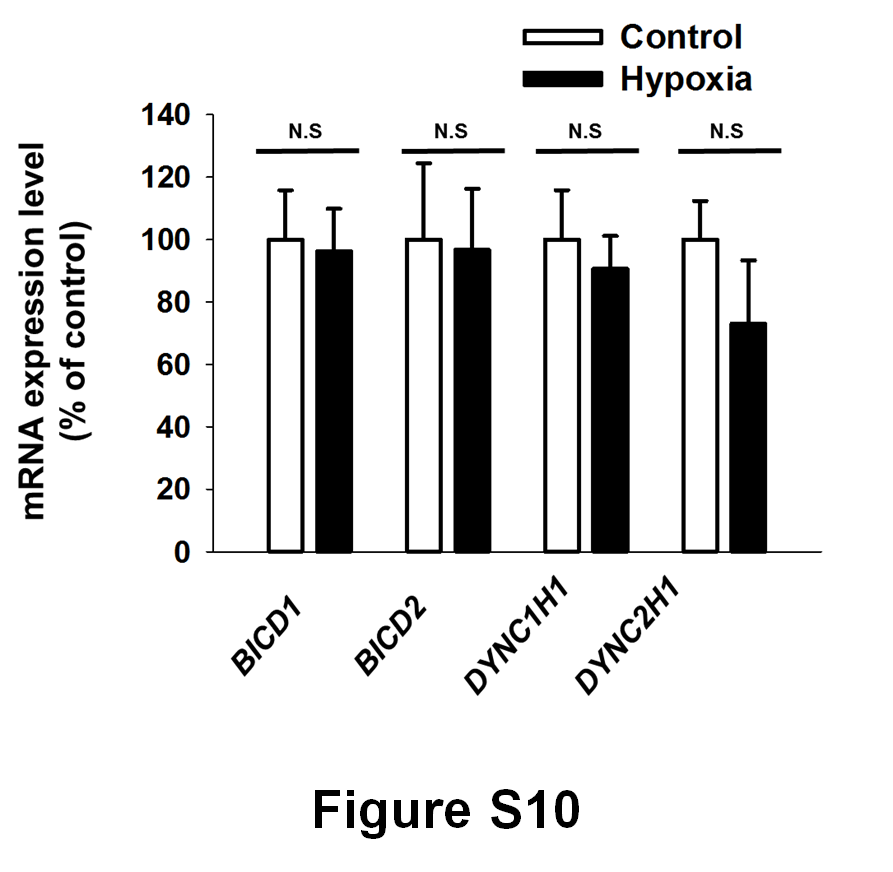

Supplement: Supplementary file 11 — Supplementary figure S10 [file 41418_2018_241_MOESM11_ESM.tif]

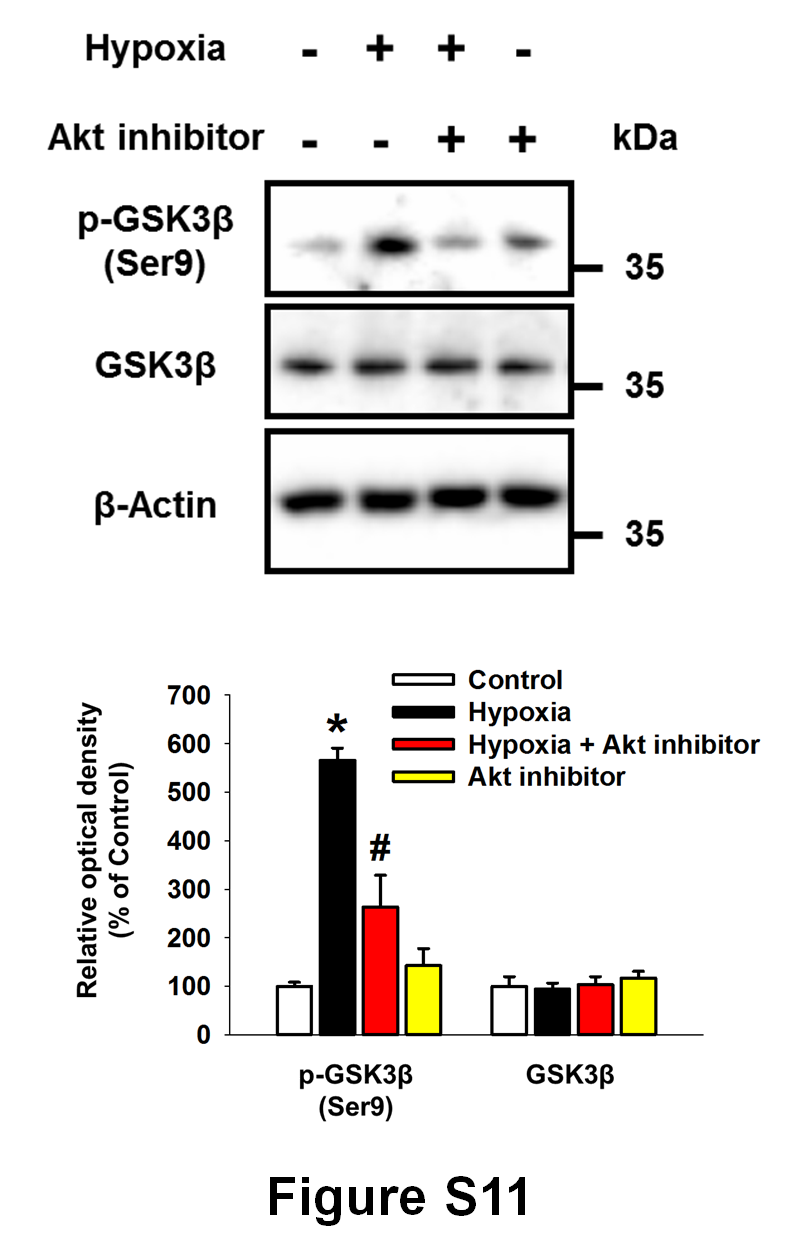

Supplement: Supplementary file 12 — Supplementary figure S11 [file 41418_2018_241_MOESM12_ESM.tif]

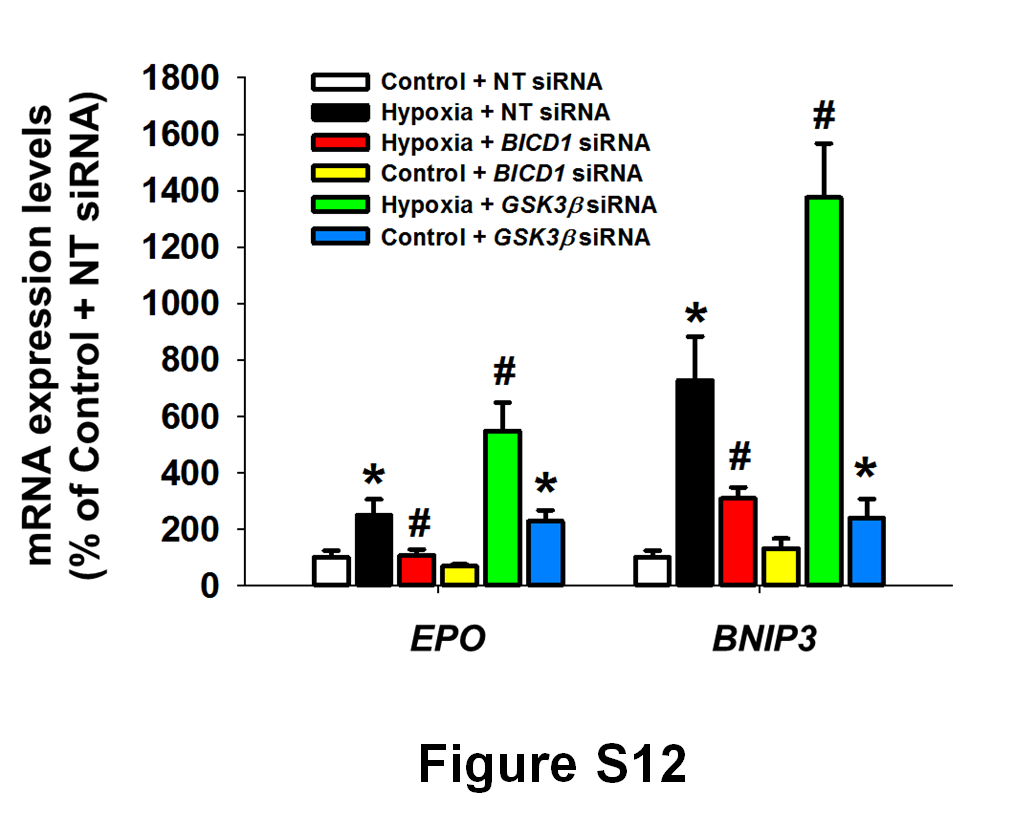

Supplement: Supplementary file 13 — Supplementary figure S12 [file 41418_2018_241_MOESM13_ESM.tif]

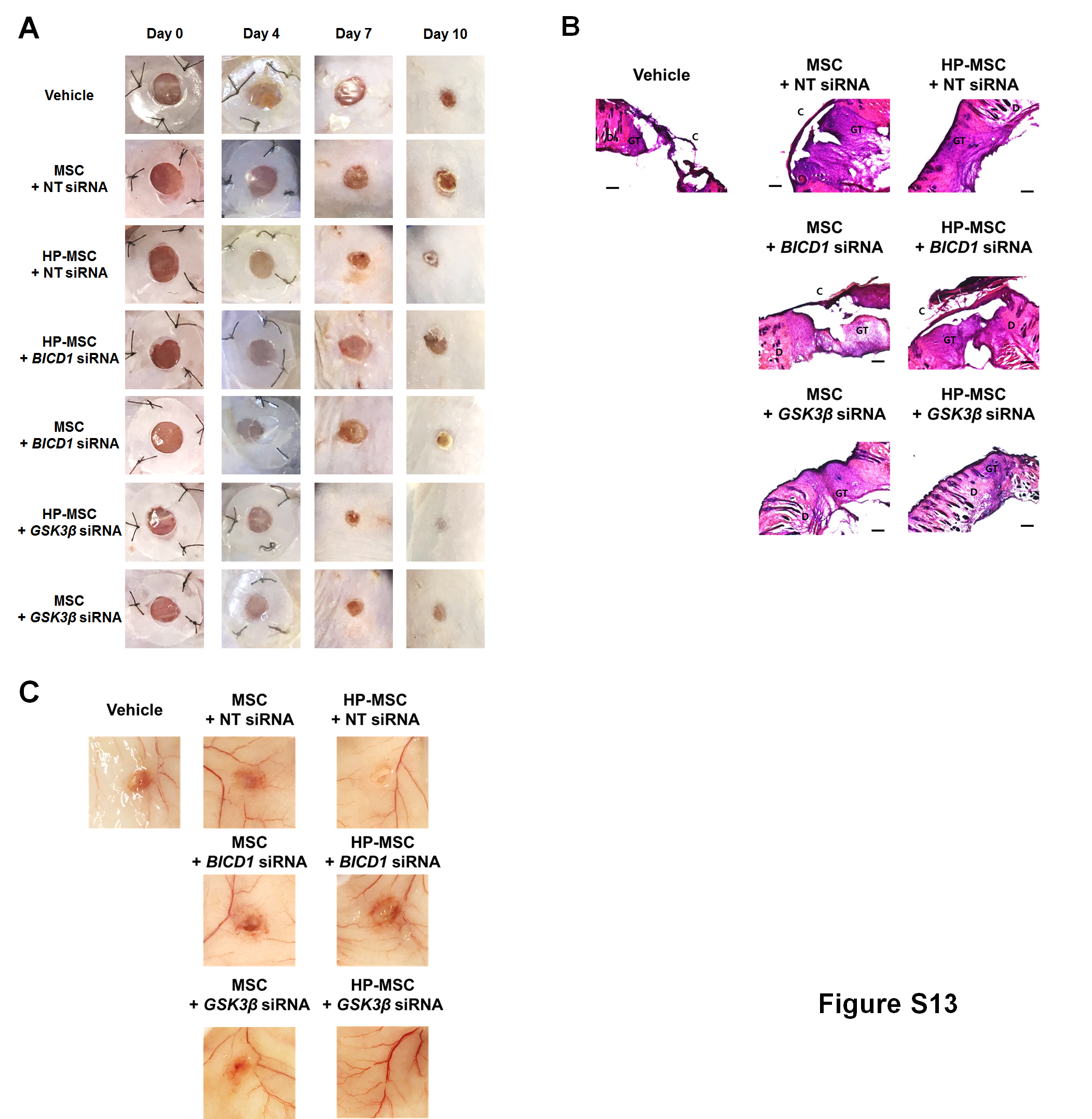

Supplement: Supplementary file 14 — Supplementary figure S13 [file 41418_2018_241_MOESM14_ESM.tif]

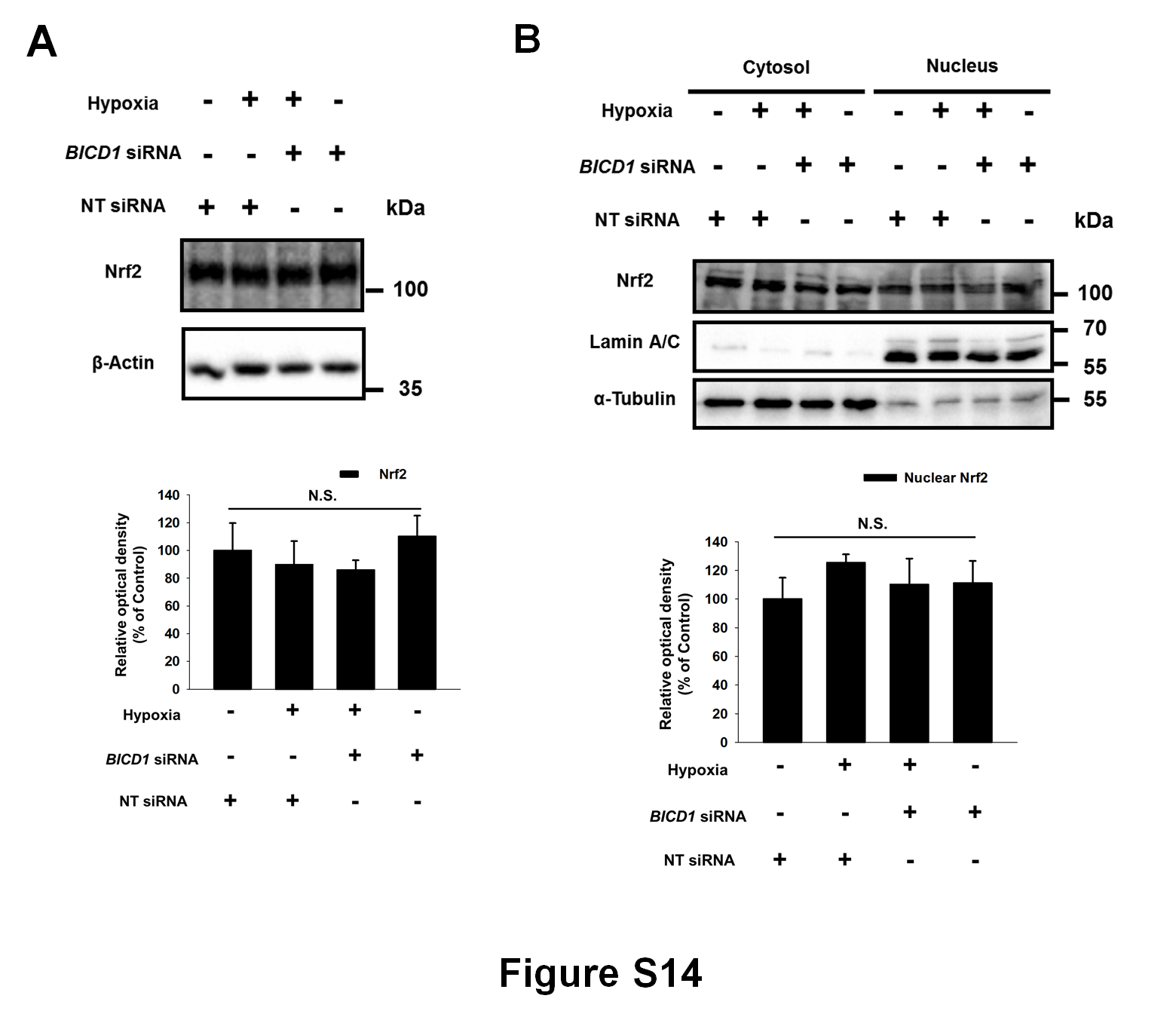

Supplement: Supplementary file 15 — Supplementary figure S14 [file 41418_2018_241_MOESM15_ESM.tif]
