# Supplementary material for: BICD1 mediates HIF1α nuclear translocation in mesenchymal stem cells during hypoxia adaptation
Source: Cell Death Differ. 2018 Nov 21;26(9):1716–34. doi: 10.1038/s41418-018-0241-1 (PMC6748134; doi:10.1038/s41418-018-0241-1)
Supplement: Supplementary file 16 — Supplementary tables [file 41418_2018_241_MOESM16_ESM.docx]

**Supplementary tables**

**Sequences of siRNAs used for gene silencing**

| Target gene | Sequence | Supplier |
| --- | --- | --- |
| *BICD1* | CUGAACACUUUGUUACGAA  UUCGUAACAAAGUGUUCAG | Bioneer |
|  | GAGGAUGGGAGUGAACCAA  UUGGUUCACUCCCAUCCUC |  |
|  | GCUAGGGAAGUUGAUUCCA  UGGAAUCAACUUCCCUAGC |  |
| *BICD2* | GGUAAAGAGUCACAUUGGU  ACCAAUGUGACUCUUUACC | Bioneer |
|  | GACGAGAAGAAGACGCUGA  UCAGCGUCUUCUUCUCGUC |  |
|  | GCUCAGCUAAGUGUAUCCA  UGGAUACACUUAGCUGAGC |  |
| *GSK3β* | GACACUAAAGUGAUUGGAA  UUCCAAUCACUUCAGUGUC | Bioneer |
|  | GACACUAUAGUCGAGCCAA  UUGGCUCGACUAUAGUGUC |  |
|  | CACUGAUUAUACCUCUAGU  ACUAGAGGUAUAAUCAGUG |  |
| Non-targeting (NT) | UAGCGACUAAACACAUCAA | Dharmacon |
|  | UAAGGCUAUGAAGAGAUAC |  |
|  | AUGUAUUGGCCUGUAUUAG |  |
|  | AUGAACGUGAAUUGCUCAA |  |

**Table S2. Sequences of primers used for RT-PCR and real-time PCR**

| Gene | Identification | Sequence (5'-3') | Size (bp) |
| --- | --- | --- | --- |
| *HK1* | Sense | GCTCTCCGATGAAACTCTCATAG | 121 |
|  | Antisense | GGACCTTACGAATGTTGGCAA |  |
| *LDHA* | Sense | ACGTCAGCAAGAGGGAGAAA | 191 |
|  | Antisense | CGCTTCCAATAACACGGTTT |  |
| *G6PD* | Sense | CGAGGCCGTCACCAAGAAC | 166 |
|  | Antisense | GTAGTGGTCGATGCGGTAGA |  |
| *BICD1* | Sense | TCCATCCACCGGAAGGTTG | 127 |
|  | Antisense | GGCTCTGTTTCAGCTCGTTC |  |
| *BICD2* | Sense | CGGAGCGCGAACAGAAGAA | 123 |
|  | Antisense | CAGCATCGTCACTGAACTTGA |  |
| *DYNC1H1* | Sense | GCTAAGAGCTGTTATCGTCAGG | 99 |
|  | Antisense | GCCACTTTCATATCTTGGGGTTC |  |
| *DYNC2H1* | Sense | ACGGCAAGTATTCGCACCAAT | 174 |
|  | Antisense | AGGATACCTCGTGTGTCATCTT |  |
| *ACTB* | Sense | AACCGCGAGAAGATGACC | 351 |
|  | Antisense | AGCAGCCGTGGCCATCTC |  |
| *EPO* | Sense | AAGCCATCTCCCCTCCAGAT | 161 |
|  | Antisense | CACACCTGGTCATCTGTCCC |  |
| *BNIP3* | Sense | GCCATCGGATTGGGGATCTAT | 150 |
|  | Antisense | GCCACCCCAGGATCTAACAG |  |
| *18S rRNA* | Sense | GGCCGTTCTTAGTTGGTGGA | 183 |
|  | Antisense | CCCGGACATCTAAGGGCATC |  |

**Table S3. Scoring of histological changes in skin wound healing**

| **Score** | **Re-epithelialization** |
| --- | --- |
| **0** | Absence of epithelial proliferation in > 70 % of the tissue |
| **1** | Poor epidermal organization in > 60 % of the tissue |
| **2** | Incomplete epidermal organization in > 40 % of the tissue |
| **3** | Moderate epithelial proliferation in > 60 % of the tissue |
| **4** | Complete epidermal remodeling in > 80 % of the tissue |
